# Brownmillerite Ca_2_Fe_2_O_5_: structural, optical and photoelectrochemical properties for efficient visible-light-driven dye degradation

**DOI:** 10.1039/d6ra04180a

**Published:** 2026-09-30

**Authors:** F. Saib, A. Almansba, L. A. Haouchine, F. M. Laoui, N. Drai, F. Touahra, S. Zemmache, M. Kebir, O. Arous, D. Lerari, K. Bachari, M. Trari

**Affiliations:** a Scientific and Technical Research Centre in Physico-chemical Analysis (C.R.A.P.C) BP384, Bou-Ismail, RP 42004 Tipaza Algeria saib.dz@gmail.com saib.faouzi@crapc.dz; b Laboratory of Industrial Process Engineering Sciences, University of Sciences and Technology Houari Boumediene BP 32, El-Alia, Bab-Ezzouar Algiers 16111 Algeria; c Crystallography-Thermodynamics Laboratory, Faculty of Chemistry USTHB, BP 32 16111 Algiers Algeria; d Laboratory of Hydrometallurgy and Inorganic Molecular Chemistry, Faculty of Chemistry USTHB, PO11Box 32 El Alia 16111 Algiers Algeria; e Unité de Recherche en Analyses Physico-Chimiques des Milieux Fluides et Sols – (URAPC-MFS/CRAPC) 11, Chemin Doudou Mokhtar, Ben Aknoun Alger 16100 Algeria; f Laboratory of Storage and Valorization of Renewable Energies, Faculty of Chemistry USTHB, BP 32 16111 Algiers Algeria

## Abstract

The main objective of this study was to investigate the structural, optical, electrochemical, and photocatalytic properties of brownmillerite Ca_2_Fe_2_O_5_ synthesized *via* the nitrate route. Comprehensive structural, morphological, and surface characterization experiments were performed using X-ray diffraction (XRD), scanning electron microscopy coupled with energy-dispersive X-ray spectroscopy (SEM-EDX), and X-ray photoelectron spectroscopy (XPS). The results confirmed the formation of phase-pure orthorhombic brownmillerite with a homogeneous elemental distribution and expected surface chemical states (Ca^2+^, Fe^3+^, and lattice oxygen). The material exhibited a relatively low specific surface area (∼8 m^2^ g^−1^). Diffuse reflectance spectroscopy revealed a direct optical band gap (*E*_g_) of 1.92 eV, attributed to the d–d transition of the Fe^3+^: 3d orbital in 6-fold coordination, enabling efficient utilization of visible solar radiation, determined by diffuse reflectance spectroscopy. The capacitance–potential (*C*^−2^*vs.* potential) characteristic in a Na_2_SO_4_ (0.1 M) solution revealed p-type conduction, with a flat band potential (*E*_fb_) of 0.21 V. The current density–potential (*J*–*E*) profile demonstrates electrochemical oxygen intercalation. The semicircle in the Electrochemical Impedance Spectroscopy is ascribed to the bulk contribution. Electrical conductivity measurements revealed a thermally activated conduction mechanism governed by small-polaron hopping, with an activation energy (*E*_a_) of 0.31 eV, indicating favorable charge transport within the brownmillerite structure. The conduction band potential, being more negative than the O_2_/O_2_˙^−^ redox level, supports the feasibility of photoinduced water reduction. Owing to its high chemical stability over a wide pH range (5–12), Ca_2_Fe_2_O_5_ demonstrated efficient photocatalytic activity for the degradation of methylene blue under visible light, highlighting its potential for environmental remediation and sustainable photocatalytic applications.

## Abstract 2

The main objective of this study was to investigate the structural, optical, electrochemical, and photocatalytic properties of brownmillerite Ca_2_Fe_2_O_5_ synthesized *via* the nitrate route.

## Graphical abstract

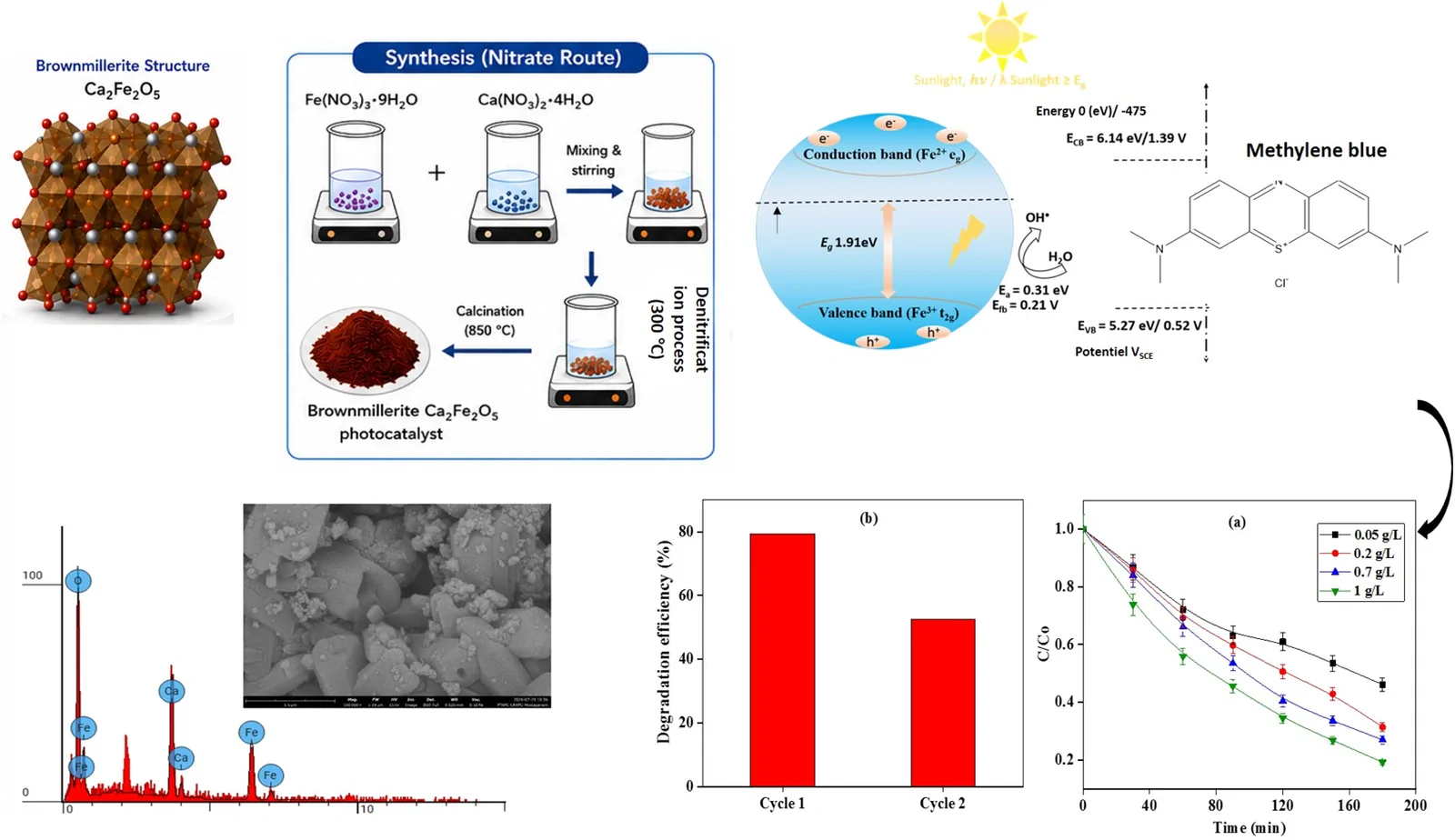

## 1 Introduction

Ternary oxides are widely utilized in solar energy conversion due to their low cost, chemical stability, and ease of synthesis.^1–5^ Therefore, they continue to garner increasing attention. Consequently, the quest for novel photocatalysts has centered on inorganic semiconductors (SCs) for the photoelectrochemical (PEC) conversion of light to usable energy.^6–10^ However, perovskite-derived compounds are still favored as electrocatalysts and photocatalysts.^11,12^ Brownmillerites, which can be represented by the general formula A_2_B_2_O_5_, exhibit high ionic conductivity owing to their layered structures.^13,14^ They are used in Li-ion batteries, photocatalysis, and a variety of other applications.^15,16^ Structurally, brownmillerites are closely related to perovskites (ABO_3_) but differ in the ordered arrangement of oxygen vacancies that produces alternating layers of metal cations in octahedral and tetrahedral coordination environments.^17,18^ This structural ordering facilitates oxygen diffusion and contributes to mixed-valence behavior at the transition-metal sites. Such frameworks can host 3d cations with variable oxidation states and accommodate a wide range of point defects, where the photocurrent originates from electronic excitation from the valence band to the acceptor levels.^19,20^ Most ferrite oxides display semiconducting properties, including visible-light absorption with a bandgap energy below 2.2 eV and a cathodic conduction band, which are key characteristics for efficient photocatalysis.^21–23^ Their relatively low electron affinity promotes efficient separation of photogenerated electron–hole (e^−^/h^+^) pairs, while an excessively wide optical gap remains the main limitation for photoelectrochemical energy conversion.^24–26^ Ca_2_Fe_2_O_5_ compound is an anion-deficient brownmillerite oxide with a narrow band gap and is composed of abundant, inexpensive, and environmentally benign elements (Ca and Fe).^1,11,12^ It remains stable below pH ≈ 13, exhibits p-type behavior with a cathodic conduction band, and thus acts as a photocatalyst with a high reducing power.^1,27^ Methylene blue (MB) dye was selected as the model organic pollutant because it is one of the most extensively used cationic dyes for evaluating the photocatalytic performance of visible-light-responsive semiconductors.^28^ Its high chemical stability, strong absorption band in the visible region (*λ*_max_ ≈ 664 nm), and well-established degradation pathway make it an excellent probe molecule for investigating photocatalytic efficiency and interfacial charge-transfer processes. Moreover, MB is widely employed in biological applications and in the textile, paper, and printing industries.^29^ Therefore, it is frequently detected in industrial wastewater owing to its persistence and potential environmental impact.^30^ Against this background, its degradation is an environmentally relevant benchmark for assessing the performance of photocatalytic materials. These properties make Ca_2_Fe_2_O_5_ particularly promising for visible-light-driven redox processes, such as dye degradation and hydrogen evolution.^1,11,12,31^ From an environmental standpoint, there is an urgent need to treat and decontaminate textile wastewater before it is discharged into natural water bodies. Therefore, the search for cost-effective and energy-efficient materials capable of harvesting a large fraction of the solar spectrum is of great importance for sustainable environmental remediation. Although several studies have separately investigated the dielectric, electrochemical, photoelectrochemical, or photocatalytic properties of Ca_2_Fe_2_O_5_, comprehensive studies establishing the relationships among these properties within a single investigation remain scarce.^32,33^ Though Ca_2_Fe_2_O_5_ has been investigated for photoelectrochemical hydrogen production under visible-light irradiation,^12^ a comprehensive understanding of its photocatalytic degradation behavior toward organic pollutants and its relationship with the electronic structure remains limited.^34,35^ In the present work, brownmillerite Ca_2_Fe_2_O_5_ was investigated as a visible-light-responsive photocatalyst for MB dye degradation. Particular emphasis was placed on correlating the crystal structure, optical properties, electrical conductivity, Mott–Schottky analysis, cyclic voltammetry, electrochemical impedance spectroscopy, and photocatalytic activity. In addition, the effects of catalyst dosage and solution pH on the photocatalytic performance, as well as the reproducibility of the photocatalytic process, were systematically investigated. This integrated approach provides deeper insight into the charge-transfer processes governing the visible-light photocatalytic performance of Ca_2_Fe_2_O_5_ and contributes to a better understanding of the structure–property–performance relationships in brownmillerite photocatalysts.

## 2 Experimental

The Ca_2_Fe_2_O_5_ compound was synthesized using the nitrate route, where stoichiometric amounts of Fe(NO_3_)_3_·9H_2_O (Biochem Chemopharma, 99.5%) and Ca(NO_3_)_2_·4H_2_O (Panreac, 98%) were dissolved in deionized water to obtain clear solutions. The homogeneous solution was gently dehydrated under magnetic stirring to prevent splashing and deviations from the intended stoichiometry. The denitrification process was performed at 300 °C in a ventilated environment until the complete removal of NO_*x*_ fumes. The resulting powder was ground and subsequently calcined at 850 °C (10 °C min^−1^ for 8 h) in air, with intermittent re-grinding to enhance homogeneity. The obtained powder was pressed into pellets (*∅* = 13 mm, thickness ≈ 0.6 mm) under a pressure of 5 kbar, followed by annealing at 850 °C to ensure their crystallinity. Thermal analysis (TG-DTA) was performed using a PerkinElmer STA 6000 instrument at a heating rate of 10 °C min^−1^. X-ray diffraction (XRD) patterns were recorded to confirm single-phase formation using Cu Kα radiation (*λ* = 1.5406 Å), with a scan rate of 2° min^−1^ in the 2*θ* range of 5–80°. The specific surface area (*S*_a_) was determined by the BET method using an ASAP 2010 Micromeritics analyzer, with nitrogen gas as the adsorbate at 77 K. Scanning Electron Microscopy (SEM) images were obtained using a Quanta 250 microscope to examine the surface morphology of the synthesized powder. UV-visible diffuse reflectance spectra were recorded with a JASCO V-650 spectrophotometer in the wavelength range of 400–800 nm.

The electrical conductivity (*σ*) was measured using a custom-built two-probe apparatus in the temperature range of 300–480 K. For ohmic contact, copper wires were attached to the pellet's surface using silver paste, and the assembly was sealed inside a glass tube with Araldite epoxy to prepare the working electrode (WE, 1.32 cm^2^). The photoelectrochemical measurements were carried out in a double-walled glass cell containing a 0.1 M Na_2_SO_4_ electrolyte and a Pt auxiliary electrode. The WE potential was controlled using a VersaSTAT 3 potentiostat/galvanostat, referenced to a saturated calomel electrode (SCE). Current–potential (*J*–*E*) curves were recorded in the dark and under visible-light illumination provided by a 200 W tungsten lamp. The capacitance–potential (*C*–*E*) plots were measured at 10 kHz, a frequency high enough to neglect the double-layer capacitance effects.

Photocatalytic experiments were conducted under natural sunlight on clear days to assess methylene blue (MB) degradation. The UV-visible absorption spectra of MB were recorded using a JASCO V-650 spectrophotometer between 400–800 nm. The tests were performed by dispersing 1 g L^−1^ of Ca_2_Fe_2_O_5_ in the MB aqueous solution under continuous magnetic stirring to ensure a uniform suspension and optimal light exposure. All reagents used were of analytical grade, and all solutions were freshly prepared using distilled water.

## 3 Results and discussion

### 3.1 Thermal analysis

Fig. 1 shows the TG curve of the reaction mixture containing Ca and Fe nitrates. Up to ∼100 °C, the mass remained almost constant, and the small losses observed were attributed to the desorption of adsorbed water. Losses associated with iron nitrate species were not visible because they were largely removed during the pre-treatment/drying of the stoichiometric mixture at 300 °C; therefore, their contribution to the initial TG signal was minimal (literature reports decomposition in the ∼160–260 °C range).^36^ Under these conditions, the iron fraction is predominantly Fe_2_O_3_ in the presence of Ca(NO_3_)_2_.^37,38^ The main mass loss occurs in two steps. Between ∼360 and 540 °C, the mass decreased markedly, which is attributed to the decomposition of calcium nitrate, globally described as Ca(NO_3_)_2_ → CaO + NO_*x*_ + O_2_ (gas). Between ∼540 and 680 °C, the decrease continues and completes, corresponding to the final breakdown of calcium nitrate and the formation of the oxide residue.^37^ Above ∼690 °C, the TG curve displayed a plateau, indicating the stabilization of the residual mass up to 900 °C, consistent with the attainment and thermal stability of the Ca_2_Fe_2_O_5_ phase.

**Fig. 1:**
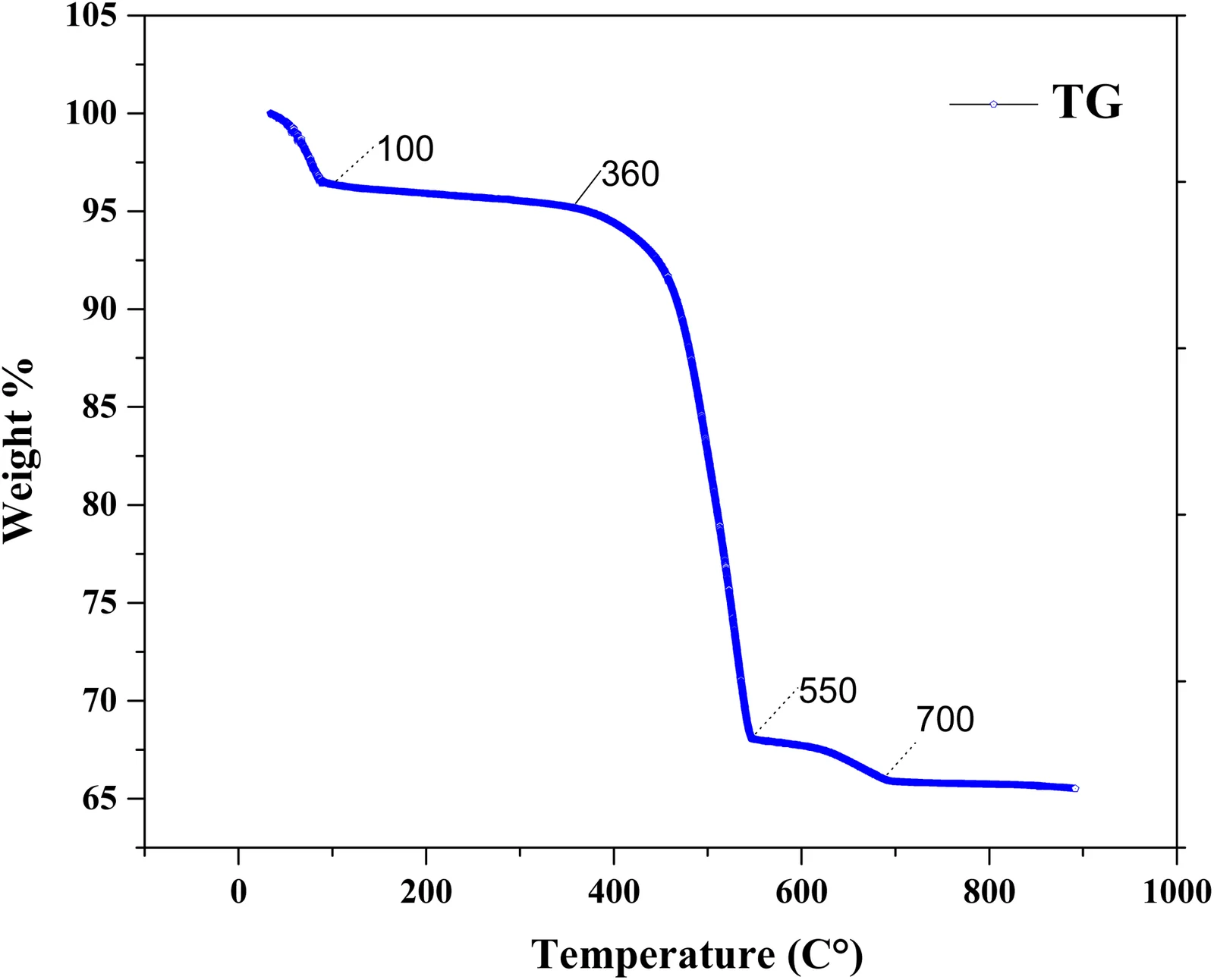
The TG plots of the nitrate mixture for the preparation of the brownmillerite Ca_2_Fe_2_O_5_.

### 3.2 X-ray diffraction

The XRD pattern of Ca_2_Fe_2_O_5_ calcined at 850 °C (Fig. 2) exhibits sharp and well-defined diffraction peaks, indicating a high degree of crystallinity of the material. According to PDF card no. 019-0222, all reflections correspond to the brownmillerite-type structure of Ca_2_Fe_2_O_5_, confirming the formation of a single-phase material without detectable secondary impurities such as CaFe_2_O_4_ and Fe_2_O_3_. The brownmillerite phase belongs to the orthorhombic system with space group *Pcmn*, in which alternating layers of corner-sharing FeO_6_ octahedra and FeO_4_ tetrahedra are arranged along the crystallographic axes.^1,2,39^ This layered configuration plays a key role in governing the electronic and ionic properties of oxides. The refined lattice parameters were *a* = 15.4290 Å, *b* = 14.7760 Å, and *c* = 5.5980 Å. These values are in good agreement with those reported in the literature for brownmillerite-type Ca_2_Fe_2_O_5_. They reflect the orthorhombic distortion inherent to the ordering of oxygen vacancies and alternation between octahedral and tetrahedral Fe sites. The relatively large *a* and *b* values compared to *c* highlight the structural anisotropy, which strongly influences the charge transport pathways. In particular, oxygen vacancy channels propagating along specific crystallographic directions facilitate ionic mobility and contribute to the p-type semiconducting behavior of this material. The characteristic brown color of the powder is consistent with visible-light absorption, as discussed in the optical study, and is directly related to the narrow band gap (*E*_g_ ≈ 1.92 eV) and the Fe^3+^ electronic configuration in a distorted crystal field. This observation further supports the assignment of the brownmillerite phase to the diffraction peaks.

**Fig. 2:**
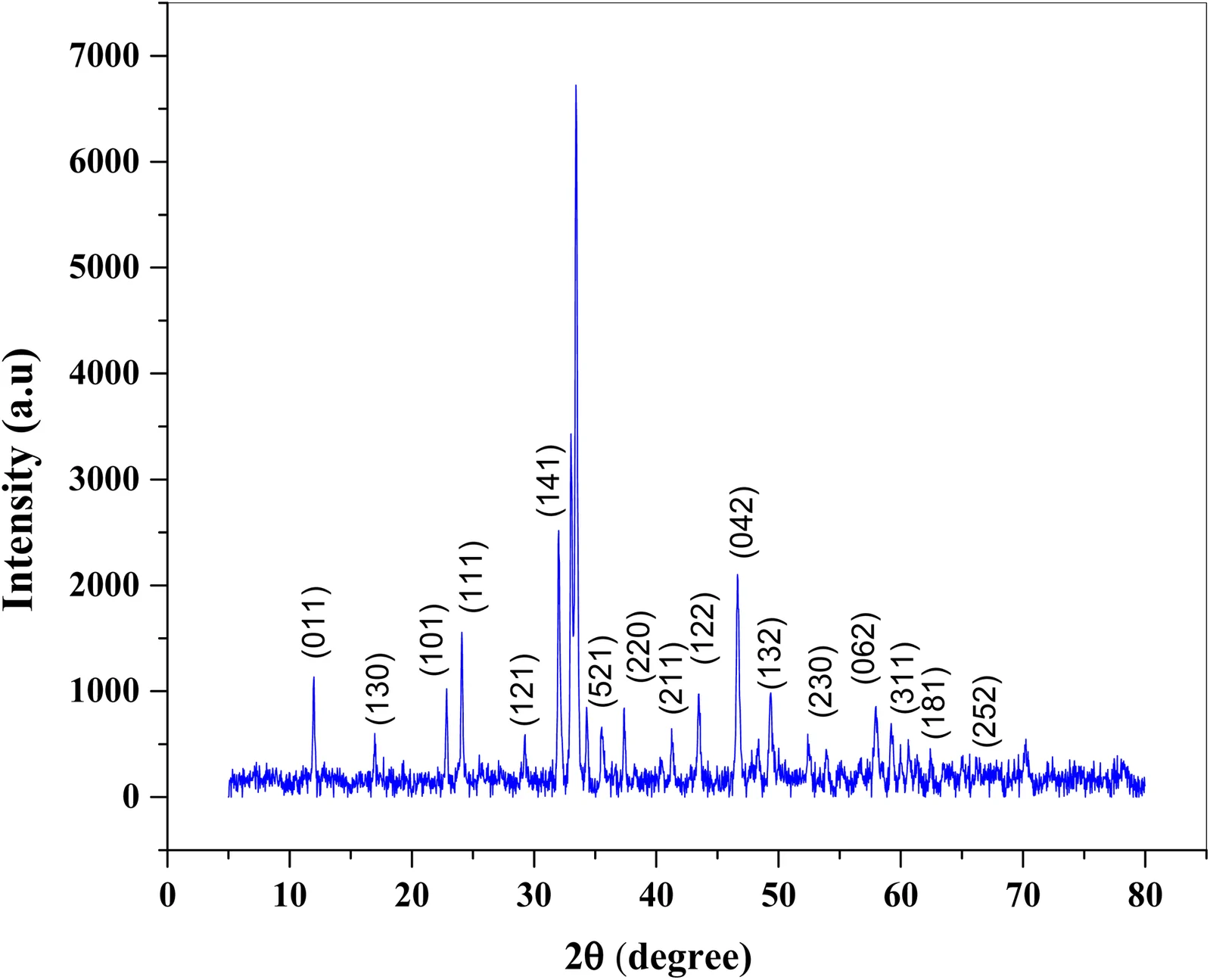
The X-ray diffraction pattern of Ca_2_Fe_2_O_5_ prepared by the nitrate route.

The specific surface area (*S*_a_ ∼8 m^2^ g^−1^) was determined using the BET method, which is consistent with the expected decrease resulting from the high calcination temperature (850 °C).

### 3.3 Scanning electron microscopy (SEM) and energy-dispersive X-ray spectroscopy (EDX)

The morphology of the synthesized Ca_2_Fe_2_O_5_ was investigated by scanning electron microscopy (SEM), and representative micrographs recorded at different magnifications are presented in Fig. 3a–d.

**Fig. 3:**
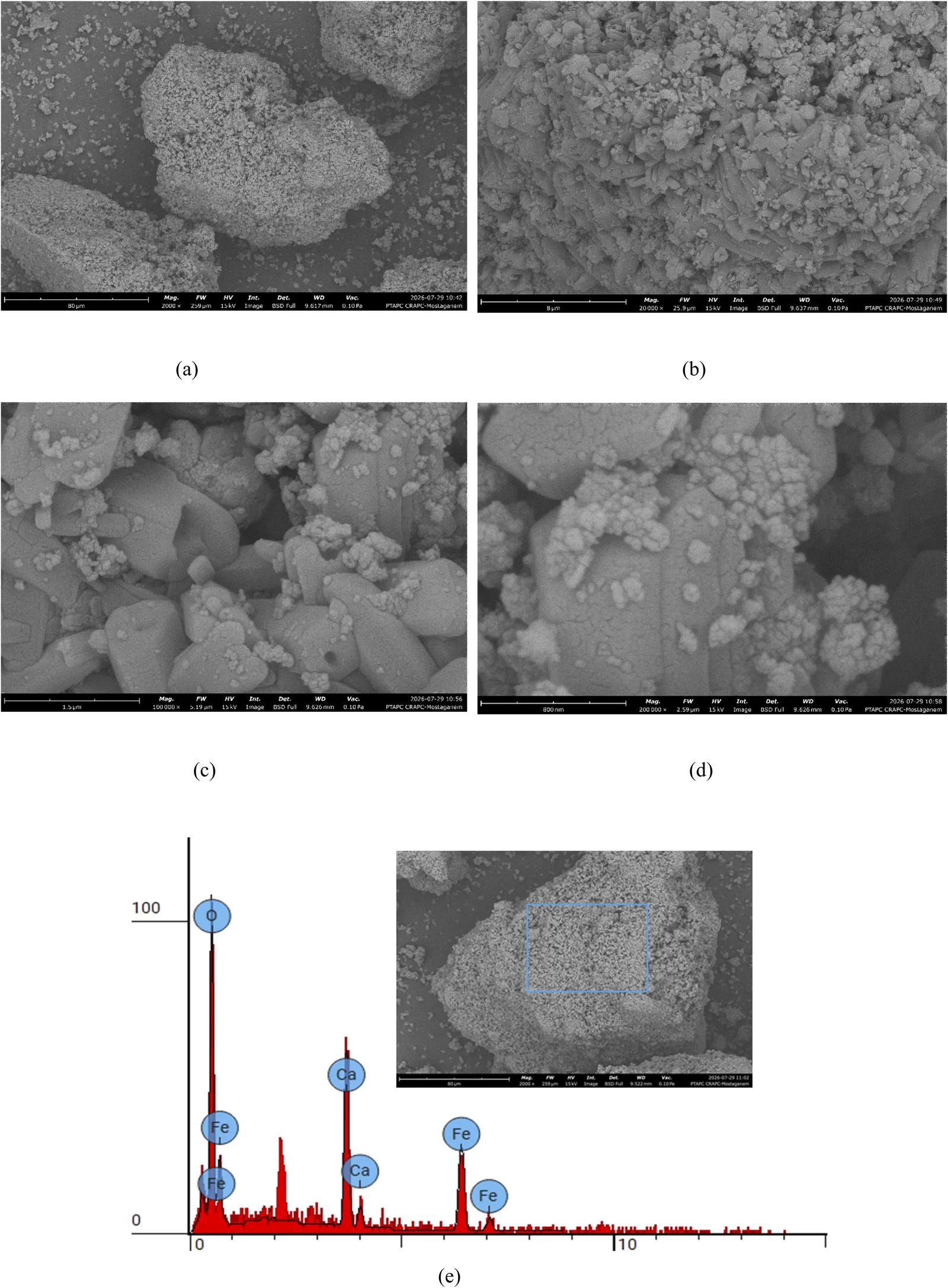
SEM micrographs of the synthesized Ca_2_Fe_2_O_5_ recorded at different magnifications: (a) 2000×, (b) 20 000×, (c) 1 00 000× and (d) 2 00 000×, (e) the EDX spectrum confirming the elemental composition and chemical purity of the photocatalyst.

At low magnification (Fig. 3a, 2000×), the synthesized powder consists of irregularly shaped micrometer-sized agglomerates distributed throughout the sample. These agglomerates are composed of numerous fine particles that are strongly interconnected, a morphology commonly observed for oxide materials synthesized *via* the nitrate route followed by high-temperature calcination.^40^ Such agglomeration can be attributed to particle coalescence and partial sintering during heat treatment. Nevertheless, the agglomerates exhibit a relatively open structure with interconnected voids, which may facilitate the diffusion of reactant molecules toward the catalyst surface during photocatalytic reactions.

At intermediate magnification (Fig. 3b, 200 00×), the agglomerates are shown to consist of densely packed crystallites exhibiting irregular polyhedral and plate-like morphologies. The crystallites are homogeneously distributed and remain strongly interconnected, forming a porous three-dimensional framework with numerous exposed grain boundaries. Such a hierarchical microstructure provides a high density of exposed grain boundaries and accessible surface-active sites, which are beneficial for photocatalytic reactions.

At higher magnification (Fig. 3c, 1 000 00×), individual crystallites become clearly distinguishable. They exhibit well-defined faceted surfaces characteristic of highly crystalline oxide particles. Numerous fine nanometric particles are observed decorating the surfaces of the larger crystallites, resulting in a hierarchical architecture with increased surface roughness. This morphology increases the surface roughness and is expected to enhance the effective contact area between the photocatalyst and pollutant molecules.

The highest-magnification image (Fig. 3d, 2 000 00×) further confirms that the surfaces of the large polyhedral crystallites are uniformly decorated with much smaller nanoparticles. The intimate contact between these nanometric particles and the larger crystallites forms a hierarchical architecture that may facilitate charge transfer across neighboring crystallites, improve the separation of photogenerated charge carriers, and provide additional adsorption sites for methylene blue molecules, thereby improving the overall photocatalytic efficiency^40,41^

To further verify the elemental composition, energy-dispersive X-ray spectroscopy (EDX) analysis was carried out, and the corresponding spectrum is presented in Fig. 3e. The quantitative elemental composition obtained from EDX is summarized in Table 1. Only calcium, iron, and oxygen were detected, confirming the absence of detectable impurity elements and demonstrating the high chemical purity of the synthesized material. The measured elemental concentrations are consistent with the expected elemental composition of Ca_2_Fe_2_O_5_. Moreover, the Ca/Fe atomic ratio calculated from the EDX data is approximately 1.05, which is in excellent agreement with the theoretical stoichiometric ratio (Ca/Fe = 1) expected for Ca_2_Fe_2_O_5_, further confirming the successful formation of the target oxide. The slight deviations from the theoretical stoichiometry can be attributed to the semi-quantitative nature of EDX analysis, the interaction volume of the electron beam, and the different X-ray generation efficiencies of light and heavy elements.^42^

**Table 1:** Quantitative elemental composition of the synthesized Ca_2_Fe_2_O_5_ determined by energy-dispersive X-ray spectroscopy (EDX)

Element number	Element symbol	Element name	Atomic conc.	Weight conc.
8	O	Oxygen	63.088	36.400
20	Ca	Calcium	18.889	27.300
26	Fe	Iron	18.024	36.300

Overall, the SEM and EDX analyses demonstrate that the synthesized Ca_2_Fe_2_O_5_ consists of chemically pure, well-crystallized agglomerates composed of interconnected polyhedral crystallites decorated with fine nanoparticles. The hierarchical porous architecture, combined with the homogeneous elemental distribution, is expected to enhance the accessibility of active sites, facilitate reactant diffusion, and improve the interaction between the catalyst surface and pollutant molecules. These structural and compositional characteristics are therefore considered to play a key role in the remarkable photocatalytic performance of Ca_2_Fe_2_O_5_ under natural solar irradiation.

### 3.4 X-ray photoelectron spectroscopy (XPS)

To investigate the surface elemental composition and the chemical states of the synthesized material, X-ray photoelectron spectroscopy (XPS) analyses were carried out. The XPS survey spectrum (Fig. 4a) reveals only the characteristic signals of calcium, iron, and oxygen, confirming the high purity of the synthesized material without any detectable impurity species. The surface atomic composition determined by XPS was 62.3 at% O, 24.4 at% Fe, and 13.3 at% Ca. Although these atomic percentages differ from the nominal bulk stoichiometry owing to the surface-sensitive nature of XPS and the different photoionization sensitivity factors of the detected elements, they nevertheless confirm the successful formation of the Ca_2_Fe_2_O_5_ phase^43–45^

**Fig. 4:**
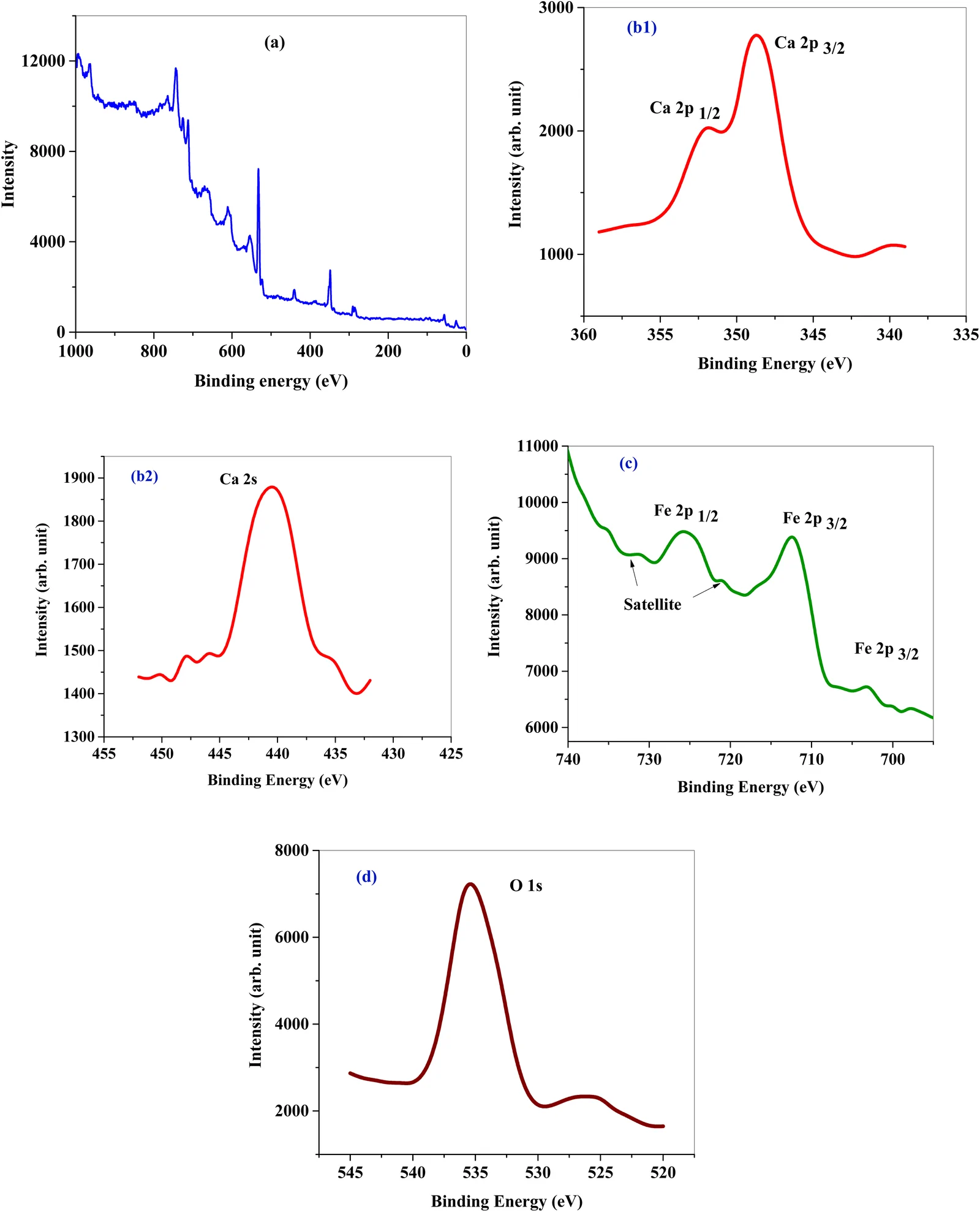
XPS characterization of the synthesized brownmillerite Ca_2_Fe_2_O_5_: (a) survey spectrum showing the elemental composition of the photocatalyst; (b1) high-resolution Ca 2p spectrum; (b2) Ca 2s core-level spectrum; (c) high-resolution Fe 2p spectrum; and (d) high-resolution O 1s spectrum.

The high-resolution Ca 2p spectrum (Fig. 4b1) exhibits the characteristic spin–orbit doublet corresponding to the Ca 2p_3/2_ (≈348.4 eV) and Ca 2p_1/2_ (≈351.4 eV) components after energy calibration. Furthermore, the Ca 2s spectrum (Fig. 4b2) displays a distinct peak located at ≈441 eV, providing additional evidence for the presence of calcium in the Ca^2+^ oxidation state. The combined Ca 2p and Ca 2s spectra confirm the successful incorporation of calcium ions into the brownmillerite crystal lattice.^43^

The high-resolution Fe 2p spectrum (Fig. 4c) displays two main photoelectron peaks located at ≈712.4 eV (Fe 2p_3/2_) and ≈725.8 eV (Fe 2p_1/2_), accompanied by characteristic shake-up satellite peaks centered at approximately 720 eV and 731 eV.^43^ The presence of these satellite features is a well-established fingerprint of Fe^3+^ species in iron oxides, confirming that iron predominantly exists in the trivalent oxidation state within the Ca_2_Fe_2_O_5_ framework. No additional components attributable to metallic iron (Fe^0^) or Fe^2+^ species were detected, indicating the complete oxidation of iron during the synthesis and the high phase purity of the prepared oxide. The high-resolution O 1s spectrum (Fig. 4d) consists of a broad asymmetric envelope centered at ≈532 eV, extending approximately from 530 to 536 eV. This broad peak is mainly assigned to lattice oxygen (O^2−^) associated with the Ca–O and Fe–O bonds characteristic of the brownmillerite structure. The slight asymmetry of the O 1s envelope also suggests a minor contribution from surface hydroxyl groups and/or adsorbed oxygen-containing species resulting from exposure of the material to ambient atmosphere.^43^

Overall, the XPS results confirm the successful formation of the brownmillerite Ca_2_Fe_2_O_5_ phase, characterized by the expected Ca^2+^ and Fe^3+^ oxidation states, in excellent agreement with the X-ray diffraction (XRD), SEM-EDX, and other structural characterization results. The coexistence of Fe^3+^ cations and lattice oxygen is expected to facilitate the transport of photogenerated charge carriers and promote the generation of reactive oxygen species (ROS), particularly ˙OH and O_2_˙^−^ radicals, under solar irradiation, thereby contributing to the excellent photocatalytic degradation performance toward MB dye. The combined XRD, SEM-EDX, and XPS analyses consistently demonstrate the successful synthesis of single-phase Ca_2_Fe_2_O_5_ with a homogeneous elemental distribution and the expected surface chemical states, providing comprehensive evidence of the structural, morphological, and surface chemical characteristics of the prepared photocatalyst.

### 3.5 Optical properties

The bandgap energy (*E*_g_) is a key parameter in photocatalysis, as it determines the fraction of the solar spectrum that can be efficiently converted into chemical or electrical energy. To evaluate the bandgap and the nature of the optical transition in Ca_2_Fe_2_O_5_, a diffuse reflectance spectrum was recorded over the 190–900 nm wavelength range. The Munk–Kubelka and Tauc methods were applied to calculate *E*_g_ using eqn (1) and (2), respectively:^46^1
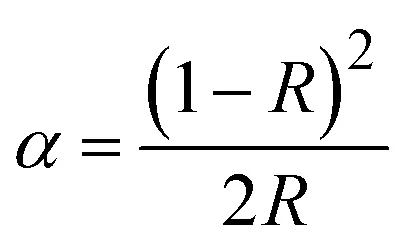

2 (*αhν*)^*m*^ = const × (*hν* − *E*_g_)

where *α* represents the optical absorption coefficient, *m* is the exponent that takes values of 2 and 1/2 for direct and indirect transitions, respectively, and *A* is a constant. Owing to their low bandgap, most ferrite oxides exhibit coloration and absorb visible light. By extrapolating the linear portion of the curve, we determined the values of the direct transitions, along with their corresponding *E*_g_ value (*E*_g_ ≈ 1.92 eV) (Fig. 5), which falls within the range typically reported for ferrite oxides (1.8–2.1 eV), confirming its ability to absorb visible light. This relatively narrow band gap is mainly attributed to the partially filled Fe^3+^ (3d^5^) configuration in the weak octahedral (Oh) crystal field, which facilitates electronic transitions in the visible region. Unlike many iron oxides, no distinct charge-transfer transition O^2−^: 2p → Fe^3+^: t_2_g was observed in the UV range, indicating that the optical response was dominated by Fe 3d intra-band transitions.

**Fig. 5:**
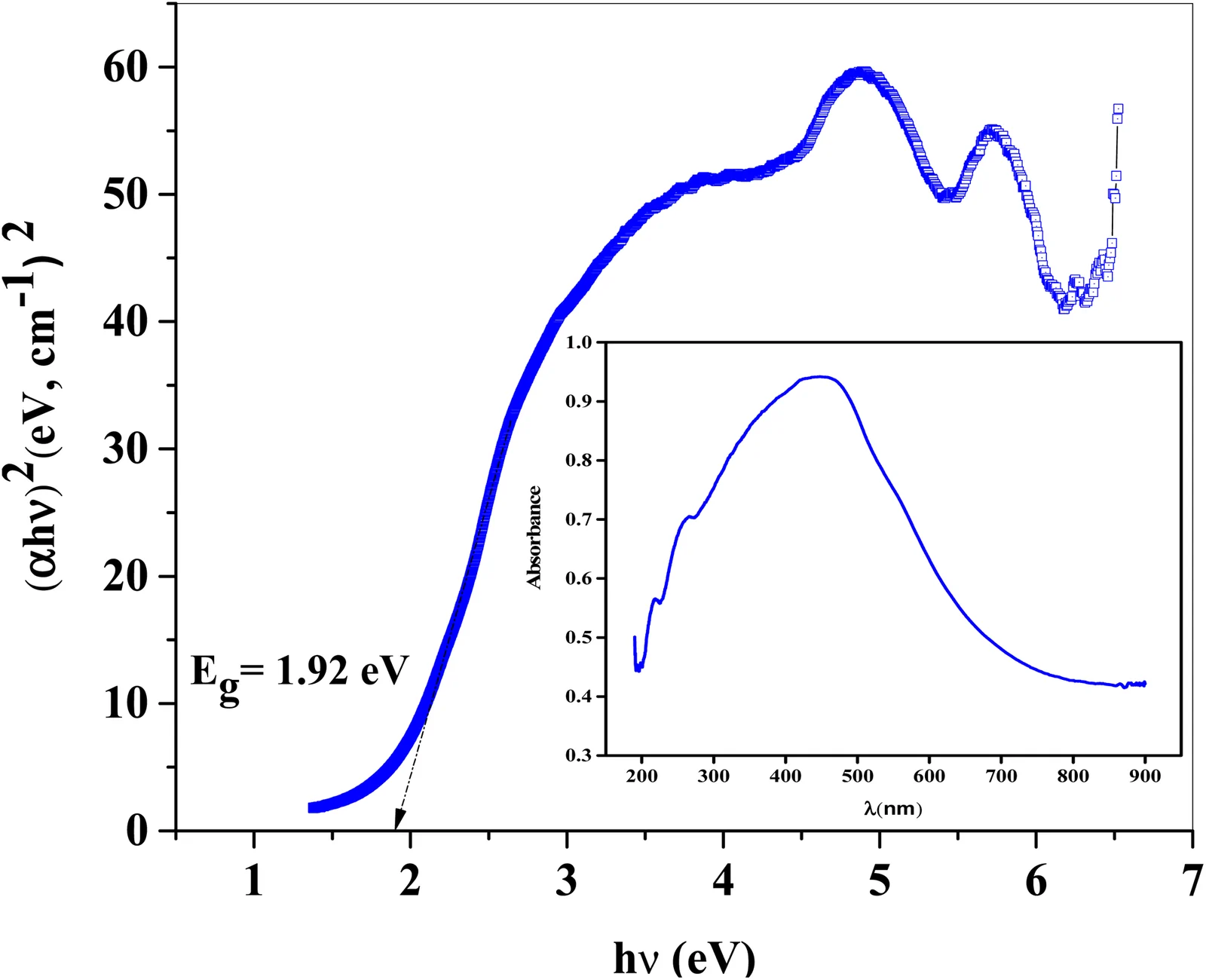
The direct optical transition of Ca_2_Fe_2_O_5_. Inset: indirect absorbance.

The electronic structure of Ca_2_Fe_2_O_5_ is further influenced by the presence of calcium. The low electronegativity of Ca^2+^ favors the stabilization of highly ionic Fe–O bonds while simultaneously enhancing their covalent character. This dual effect broadens the Fe–O bandwidth, reduces the band gap, and contributes to the observed reddish color of the material.^47,48^ From an electrical perspective, Ca_2_Fe_2_O_5_ exhibits p-type semiconducting behavior, which is closely related to the oxygen nonstoichiometry of the brownmillerite structure.^49^ The insertion of interstitial oxygen between corner-sharing FeO_6_ octahedra generates holes (h^+^) associated with Fe^3+^ cations, giving rise to small polaron conduction.^50^ To preserve charge neutrality, some Fe^3+^ is oxidized to Fe^4+^, leading to a mixed-valence Fe^3+^/Fe^4+^ state. This mixed valency is highly beneficial for charge transport because it facilitates hole hopping between neighboring Fe sites. Therefore, the observed optical and electrical properties of Ca_2_Fe_2_O_5_ can be directly correlated with the interplay between Fe–O bond covalency, oxygen defect chemistry, and polaronic conduction.

The temperature dependence of the electrical conductivity of the p-type Ca_2_Fe_2_O_5_ compound is illustrated in Fig. 6 as a plot of log *σ* (Ω^−1^ cm^−1^) *versus* 1000/*T* (K^−1^). The curve exhibits typical semiconducting behavior, where the conductivity increases with temperature, indicating a thermally activated conduction process. Linear fitting according to the Arrhenius relation, *σ* = *σ*_0_ exp(−*E*_a_/*Kt*), yields an activation energy of 0.31 eV. This relatively low activation energy suggests the easy mobility of charge carriers and confirms the semiconducting nature of the material. In such ferrite-type oxides, electrical conduction generally occurs *via* small polaron hopping between neighboring Fe^3+^ and Fe^2+^ sites.^49–51^ Therefore, the conduction mechanism can be ascribed to p-type polaronic transport, in which holes are the majority carriers. This behavior is consistent with the mixed-valence state of Fe cations in the orthorhombic Ca_2_Fe_2_O_5_ lattice, which promotes Fe^3+^ Fe^2+^ electron exchange under thermal excitation.^50,51^

**Fig. 6:**
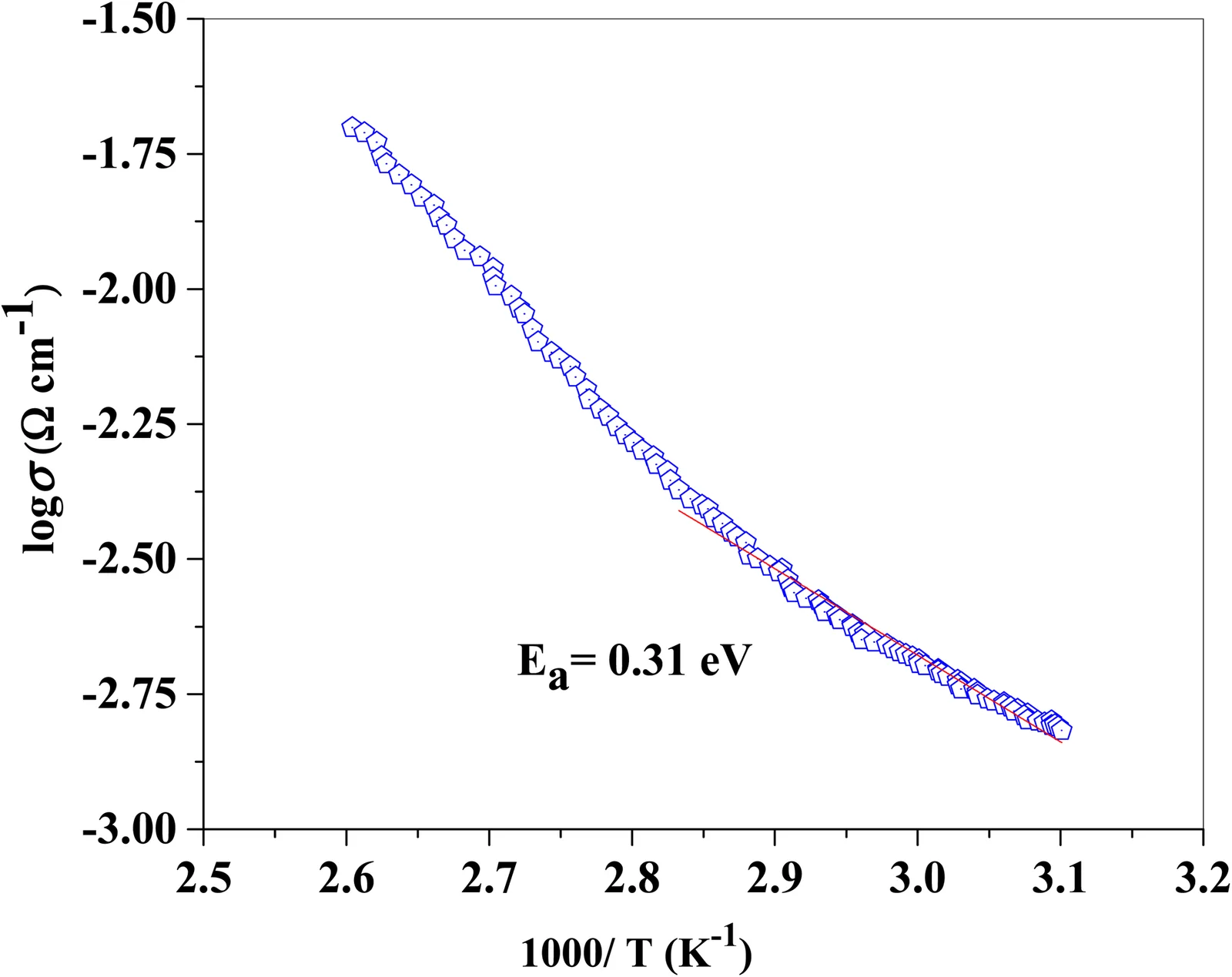
The thermal variation of the electrical conductivity of Ca_2_Fe_2_O_5._

### 3.6 Photo-electrochemistry characterization

The Mott–Schottky plot of Ca_2_Fe_2_O_5_ (Fig. 7), recorded in 0.1 M Na_2_SO_4_ solution (pH ≈ 7), displayed a negative slope, which is characteristic of a p-type semiconductor. This behavior is consistent with the electronic structure of brownmillerite Ca_2_Fe_2_O_5_, where oxygen nonstoichiometry and mixed valence states (Fe^3+^/Fe^4+^) generate hole carriers (h^+^). By extrapolating the linear portion of the curve, the flat-band potential (*E*_fb_) was determined to be approximately +0.21 V. This relatively positive value confirms the p-type conductivity and indicates that the Fermi level is closer to the valence band than to the conduction band. Such a position is favorable for oxidation reactions because the photogenerated holes in the valence band can act as strong oxidizing agents during photocatalytic processes.

**Fig. 7:**
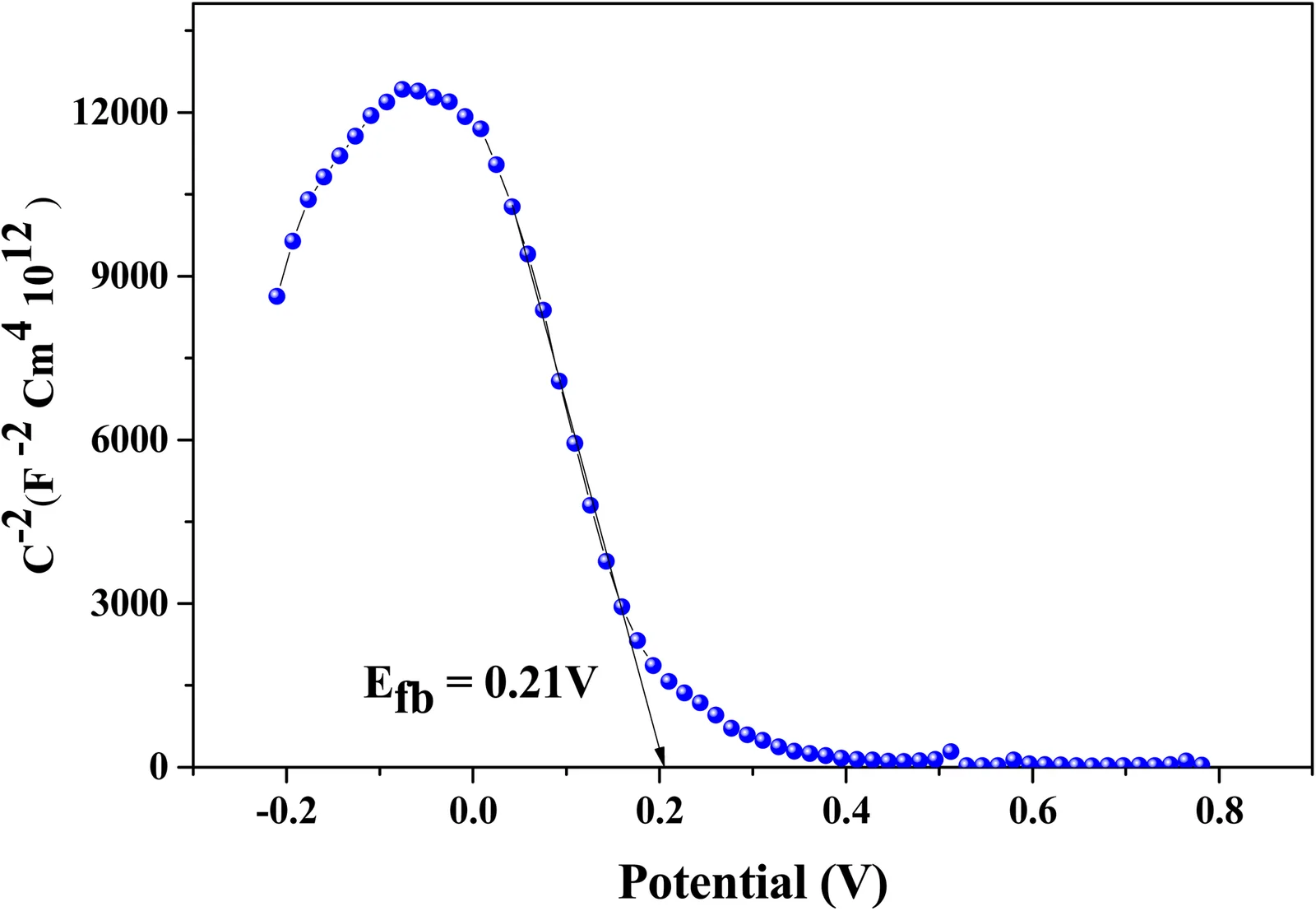
The Mott–Schottky characteristic of Ca_2_Fe_2_O_5_ in Na_2_SO_4_ solution (0.1 M, pH ∼7).

The curvature observed at more cathodic potentials (<0 V) suggests the presence of surface states or defect-related contributions, which may influence the charge transfer at the electrode/electrolyte interface. At more anodic potentials (>0.4 V), the capacitance tends to saturate, reflecting the depletion of mobile charge carriers in the semiconductor. In summary, the Mott–Schottky analysis confirmed that Ca_2_Fe_2_O_5_ behaves as a p-type semiconductor with a flat-band potential of approximately +0.21 V, consistent with hole-dominated conduction *via* a small-polaron hopping mechanism. These findings are in good agreement with the reddish coloration of the material and its narrow optical band gap (*E*_g_ ≈ 1.92 eV), further confirming that Ca_2_Fe_2_O_5_ is an efficient visible-light-absorbing semiconductor with strong potential for photocatalytic and photoelectrochemical applications.

The cyclic voltammogram of Ca_2_Fe_2_O_5_ recorded in a neutral Na_2_SO_4_ solution (0.1 M, pH ∼7) at a scan rate of 10 mV s^−1^ and in the absence of light revealed an electrochemical behavior characterized by reversible redox processes and a pseudocapacitive nature. The potentials were reported *versus* a saturated calomel electrode (SCE), and the scan direction is indicated in Fig. 8. Two main redox peaks were observed: a cathodic peak centered at approximately −0.17 V (*vs.* SCE), attributed to the reduction of Fe^3+^ to Fe^2+^, and a small anodic peak located at approximately +0.17 V (*vs.* SCE), corresponding to the reverse oxidation of Fe^2+^ to Fe^3+^. The relatively small peak-to-peak separation indicates a quasi-reversible Fe^3+^/Fe^2+^ redox process, proving the good electrochemical activity and stability of the Ca_2_Fe_2_O_5_ brownmillerite electrode. The overall closed loop of the voltammogram reflects a pseudocapacitive behavior arising from surface faradaic reactions and efficient charge transfer processes at the electrode/electrolyte interface. This behavior is consistent with the electrochemical impedance spectroscopy (EIS) results discussed in Fig. 9, which shows a relatively low charge transfer resistance and enhanced interfacial charge transport under illumination. The good agreement between the CV and EIS results corroborates the favorable charge transfer characteristics of Ca_2_Fe_2_O_5_, which are beneficial for its photoelectrochemical and photocatalytic performance.

**Fig. 8:**
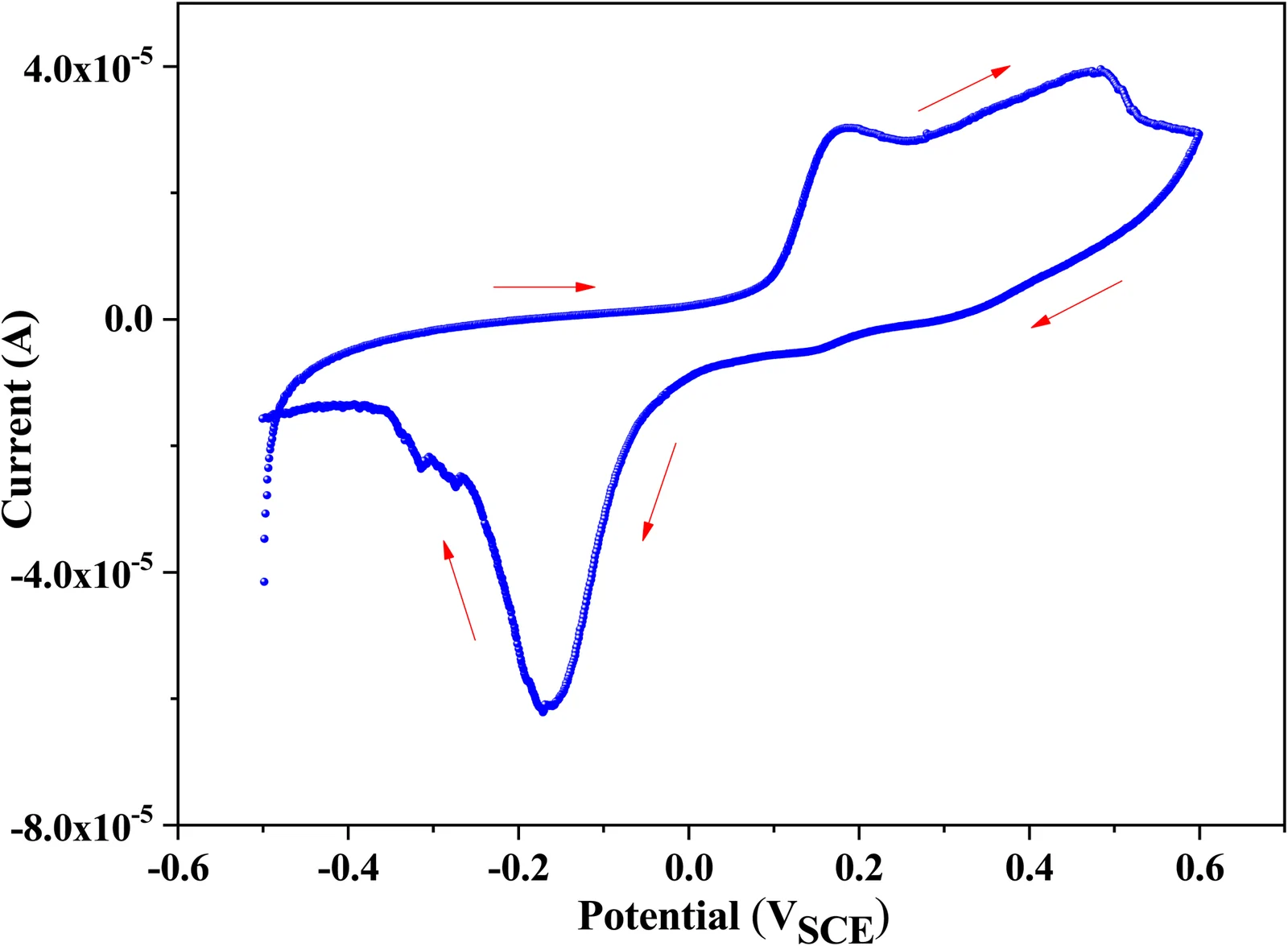
Cyclic voltammogram of Ca_2_Fe_2_O_5_ recorded in 0.1 M Na_2_SO_4_ solution (pH ≈ 7) in the dark at a scan rate of 10 mV s^−1^. Potentials are referenced to the saturated calomel electrode (SCE). Arrows indicate the scan direction.

**Fig. 9:**
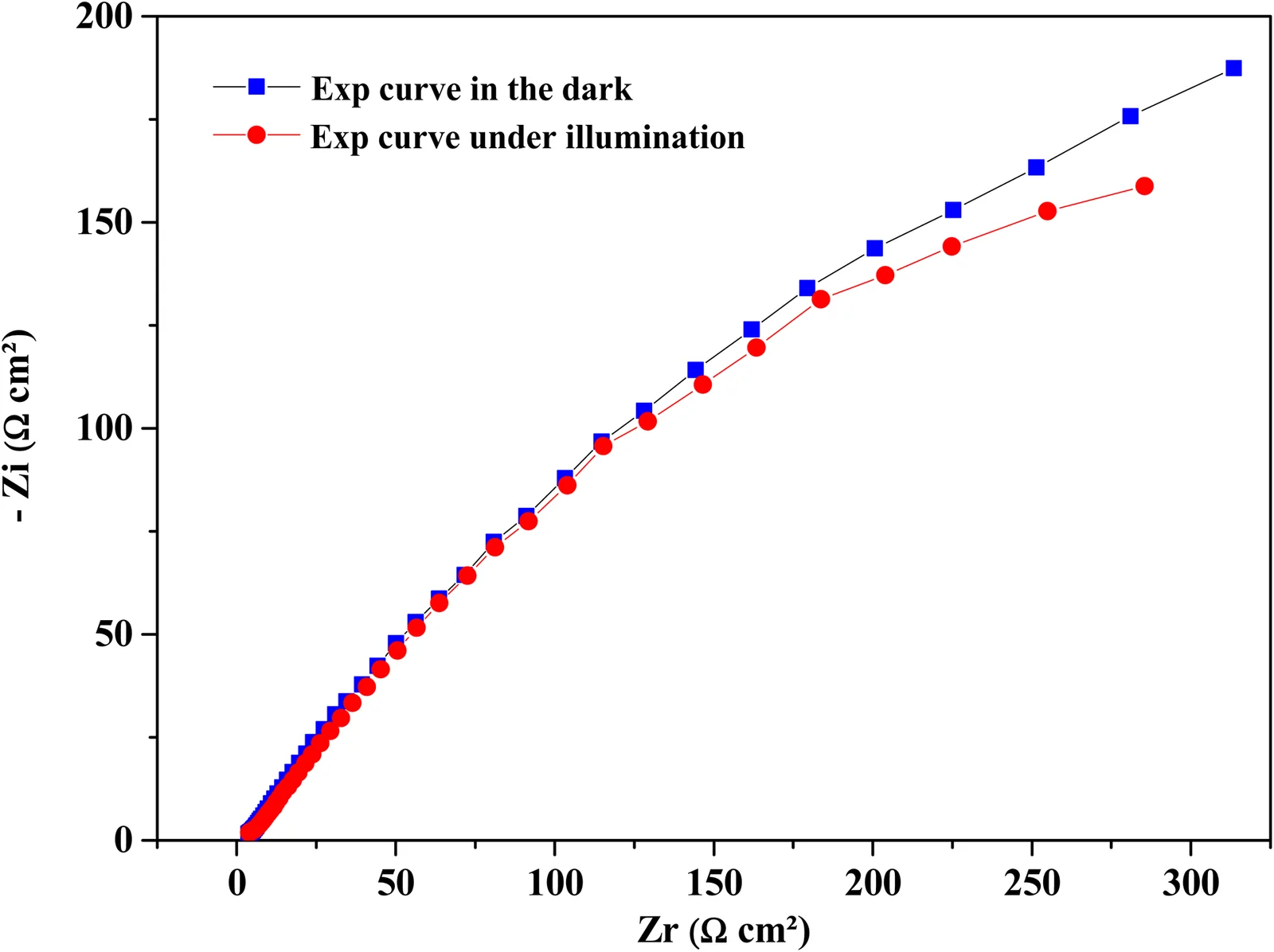
The dark and illumination Nyquist representation of Ca_2_Fe_2_O_5_ in Na_2_SO_4_ solution (0.1 M, pH ∼7).

Fig. 9 displays the Nyquist plots recorded for the Ca_2_Fe_2_O_5_ electrode in 0.1 M Na_2_SO_4_ solution (pH ≈ 7) under dark and illuminated conditions. In both cases, the impedance response can be described by a typical Randles circuit consisting of three contributions: an electrolyte resistance (*R*_s_), nearly identical in the dark and under illumination, confirming that the electrolyte is unaffected by light; a depressed semicircle associated with the parallel combination (*R*_ct_//*C*_dl_) describing charge transfer at the electrode/electrolyte interface; and an inclined straight line at ∼45° corresponding to a Warburg impedance related to ionic diffusion within the porous solid phase. The illuminated spectrum (red points) was systematically shifted toward lower real-impedance values compared to the dark spectrum (blue points). In particular, the semicircle radius decreased from ∼300 to ∼250 Ω cm^2^, corresponding to a ∼17% reduction in the charge-transfer resistance (*R*_ct_). Moreover, the Warburg branch shifted toward lower *Z*′ values, indicating that illumination enhanced ionic diffusion by reducing the internal potential gradient. This improvement in the electrochemical behavior originated from the generation of additional photoinduced charge carriers (electrons and holes), which increased the electrical conductivity of Ca_2_Fe_2_O_5_ and facilitated interfacial charge transfer.

These results are consistent with the cyclic voltammetry (CV) measurements, where the quasi-symmetric anodic and cathodic peaks associated with the Fe^3+^/Fe^2+^ redox couple indicate a quasi-reversible electron transfer process and good electrochemical reversibility. This behavior is reflected in the EIS spectra by the presence of a single depressed semicircle without additional arcs, suggesting a dominant interfacial charge transfer process. Furthermore, the pseudocapacitive behavior observed in the CV is consistent with the nonideal capacitive response (CPE-like behavior) evidenced by the depressed semicircle in the impedance spectra, highlighting the contribution of surface faradaic reactions. Overall, Fig. 9 demonstrates that illumination simultaneously decreases the charge-transfer resistance and facilitates interfacial charge transport, confirming that photogenerated charge carriers enhance the electrochemical activity of Ca_2_Fe_2_O_5_. In summary, illumination markedly enhanced the charge transport and transfer properties of Ca_2_Fe_2_O _8_ in neutral Na_2_SO_4_ electrolyte, thus confirming the beneficial effect of light on the electrochemical activity of this material.

The p-type Ca_2_Fe_2_O_5_ semiconductor exhibits distinctive electronic, electrochemical, and photocatalytic properties, highlighting its multifunctional potential for energy conversion and environmental remediation. The energy band diagram (Fig. 10) revealed that the conduction and valence band edges were located at 6.14/1.39V and 5.27/0.52 V, respectively, with an activation energy (*E*_a_) of 0.31 eV and a flat band potential (*E*_fb_) of 0.21 eV. The Fermi level is positioned close to the valence band, confirming the p-type nature of the material, in which holes are the majority carriers. Such a configuration facilitates efficient charge transfer across the semiconductor interface, which is a key factor for redox processes under light or electrochemical excitation.

**Fig. 10:**
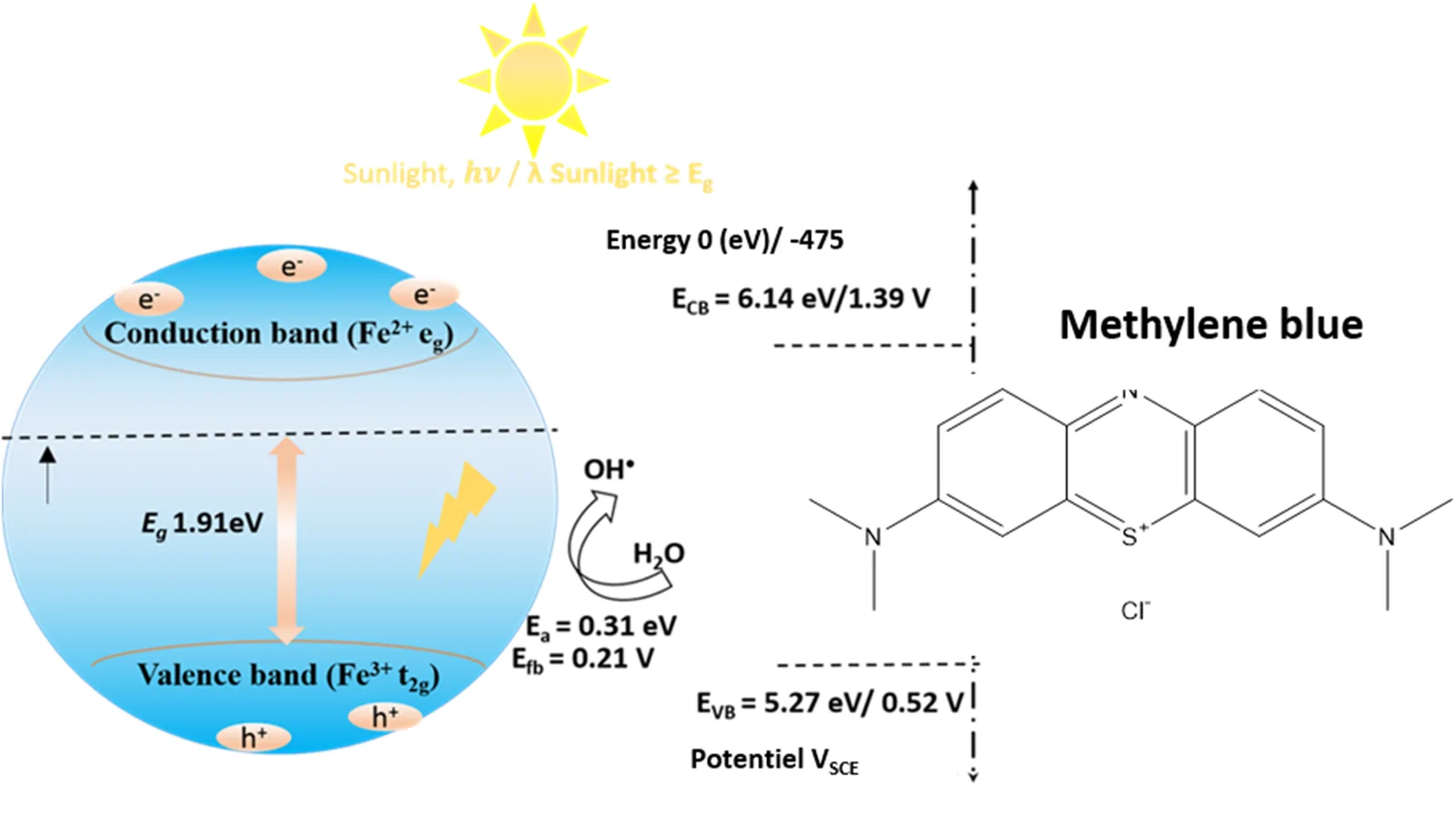
The energy diagram of the junction Ca_2_Fe_2_O_5_/Na_2_SO_4_ solution.

### 3.7 Photocatalytic test

The photocatalytic performance of Ca_2_Fe_2_O_5_ was evaluated through the degradation of MB dye under natural solar irradiation. The evolution of the normalized concentration (*C*/*C*_o_) as a function of irradiation time under different experimental conditions is presented in Fig. 10–13. In all cases, the MB concentration progressively decreased with increasing irradiation time, confirming the photocatalytic activity of the synthesized Ca_2_Fe_2_O_5_. However, the photocatalytic degradation process involves two main stages:

(i) Adsorption, where MB molecules are first adsorbed on the catalyst surface through electrostatic and van der Waals interactions; and (ii) photocatalytic oxidation, initiated once the semiconductor absorbs photons with an energy equal to or higher than its bandgap. Upon illumination, electron–hole pairs (e^−^/h^+^) are generated according to the following reaction:^52^

3 Ca_2_Fe_2_O_5_ + *hν* → e_CB_^−^ + h_VB_^+^

The photogenerated electrons in the conduction band (CB = 1.39 eV) can react with dissolved oxygen to form superoxide radicals, while the photogenerated holes in the valence band (VB = 0.52 eV) oxidize surface hydroxyl groups or adsorbed water to generate hydroxyl radicals:

4 O_2_ + e_CB_^−^ → O_2_^˙−^

5 H_2_O + h_VB_^+^ → ˙OH + H^+^

These reactive oxygen species (ROS), mainly ˙OH and O_2_˙^−^, are responsible for the oxidative degradation of MB into harmless mineralization products, according to the simplified reaction:

6 MB + ROS → CO_2_ + H_2_O + NO_3_^−^ + SO_4_^2−^

The gradual decrease in *C*/*C*_o_ with irradiation time and the linear pseudo-first-order kinetic plots obtained under different experimental conditions indicate that the degradation process follows an apparent pseudo-first-order kinetic model. These results are consistent with the favorable band structure of Ca_2_Fe_2_O_5_, which promotes efficient charge separation and transfer capabilities, thereby enhancing the generation of reactive oxygen species responsible for the photocatalytic degradation of MB.

## 4 Effect of initial solution pH

The initial solution pH plays a crucial role in heterogeneous photocatalytic processes because it influences the surface properties of the photocatalyst, the adsorption behavior of the pollutant, and the generation of reactive oxygen species (ROS). Therefore, the photocatalytic degradation of methylene blue (MB, 10 mg L^−1^) over 0.05 g of Ca_2_Fe_2_O_5_ was investigated at different initial pH values under natural solar irradiation. As shown in Fig. 11(a), the normalized concentration (*C*/*C*_o_) gradually decreased with increasing irradiation time under all investigated pH conditions, confirming the photocatalytic activity of Ca_2_Fe_2_O_5_. However, the degradation rate was strongly dependent on the initial solution pH. The corresponding degradation efficiencies after 180 min are presented in Fig. 11(b). The highest degradation efficiency (approximately 53%) was obtained at natural pH (≈6). In comparison, the degradation efficiency was approximately 25%, 43%, and 34% at pH 4, 8, and 10, respectively, while almost no significant degradation was observed at pH 2.

**Fig. 11:**
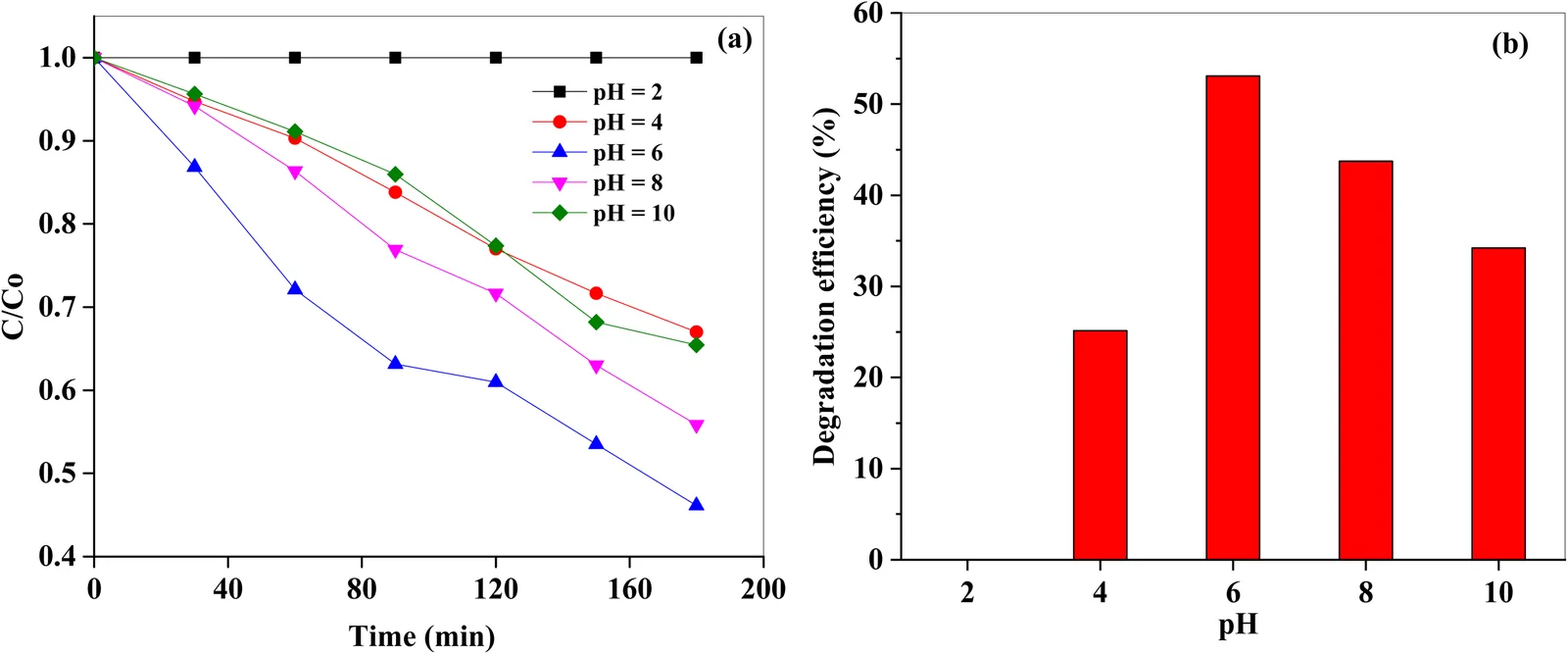
Effect of the initial solution pH on the photocatalytic degradation of 10 mg per L MB over 0.05 g of Ca_2_Fe_2_O_5_ under natural solar irradiation: (a) normalized concentration (*C*/*C*_o_) *versus* irradiation time; (b) photocatalytic degradation efficiency at different pH values.

The poor photocatalytic activity observed under strongly acidic conditions can be attributed to the high concentration of H^+^ ions, which compete with MB molecules for the catalyst surface and suppress the formation of hydroxyl radicals (˙OH), thereby reducing the oxidation efficiency. As the solution pH increased to approximately 6, a significant enhancement in photocatalytic activity was observed, indicating that these conditions provide a favorable environment for both pollutant adsorption and the generation of reactive oxygen species responsible for MB degradation.

Further increasing the solution pH to alkaline values (pH 8–10) resulted in a slight decrease in photocatalytic efficiency. Although hydroxyl ions are more abundant in alkaline media and may promote the formation of hydroxyl radicals, excessive OH^−^ ions may also compete for the catalyst surface, modify the surface reaction kinetics, and facilitate the recombination of photogenerated charge carriers, thereby limiting the overall photocatalytic performance. Consequently, natural pH (≈6) was identified as the optimum condition for MB degradation over Ca_2_Fe_2_O_5_.

Based on these results, all subsequent photocatalytic experiments investigating the effect of catalyst dosage, reaction kinetics, and catalyst reusability were performed under natural pH conditions (≈6), corresponding to the optimum operating conditions.

### 4.1 Effect of catalyst dosage

The effect of catalyst dosage on the photocatalytic degradation of MB, 10 mg L^−1^ was investigated by varying the Ca_2_Fe_2_O_5_ dosages from 0.05 to 1 g L^−1^ under natural solar irradiation at natural pH (≈6). As shown in Fig. 12(a), the photocatalytic degradation efficiency increased progressively with irradiation time for all catalyst dosages. Moreover, increasing the catalyst dosage significantly enhanced the photocatalytic degradation efficiency, while the degradation efficiency increasing from approximately 53% at 0.05 g L^−1^ to about 79% at 1 g L^−1^, as illustrated in Fig. 12(b). However, the improvement became less pronounced at higher catalyst dosages, indicating that further increasing the catalyst dosage provides only a marginal improvement in the photocatalytic performance. This behavior can be attributed to the increase in the number of available active sites with increasing catalyst dosage, which promotes the generation of reactive oxygen species (ROS) and consequently enhances the degradation of MB.^53^ However, at higher catalyst loadings, the suspension becomes increasingly turbid, resulting in a light-screening effect that reduces light penetration and limits the effective photoactivation of the catalyst.^54^ Conversely, at low catalyst dosages, the limited number of available active sites results in lower ROS generation and, consequently, reduced photocatalytic efficiency.^55^

**Fig. 12:**
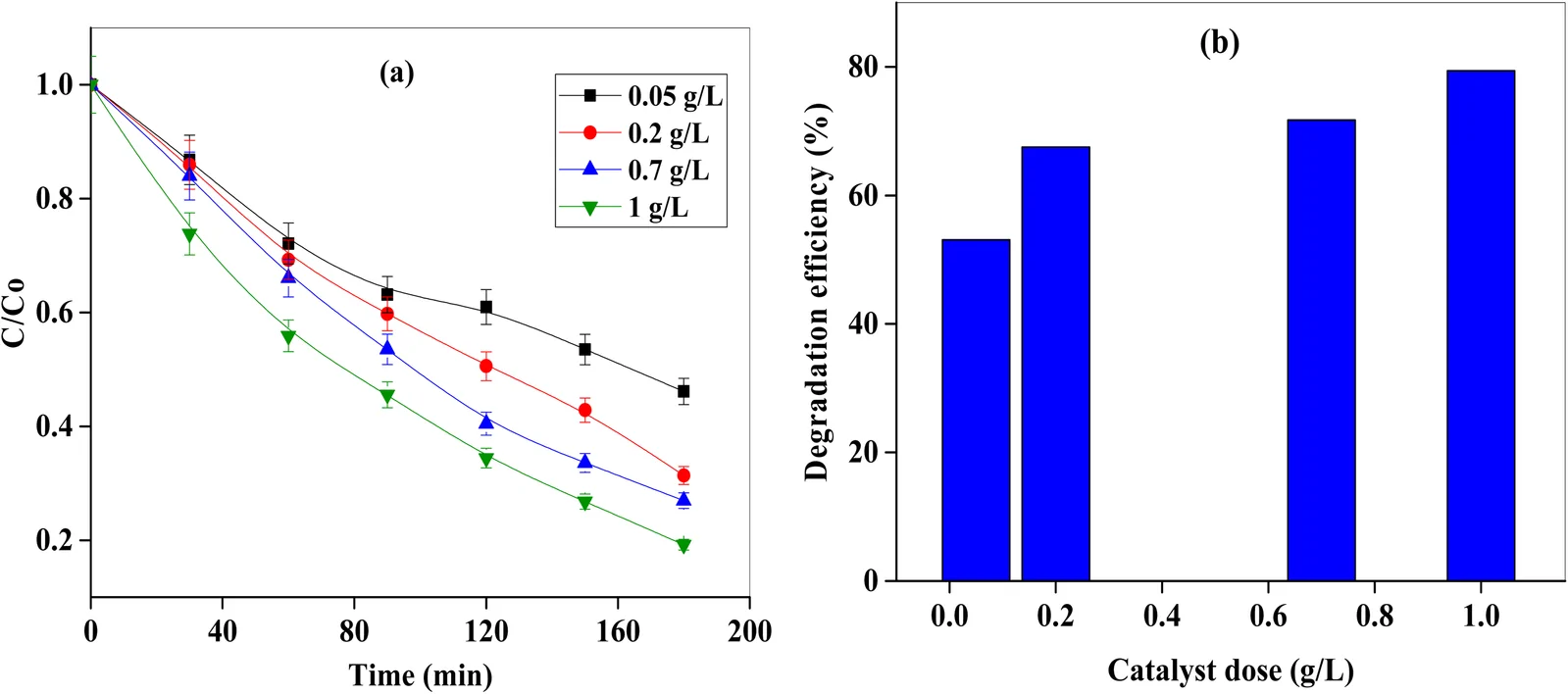
Effect of Ca_2_Fe_2_O_5_ dosage on the photocatalytic degradation of 10 mg per L MB under natural solar irradiation at free pH (≈6): (a) normalized concentration (*C*/*C*_o_) *versus* irradiation time; (b) degradation efficiency after 180 min.

### 4.2 Photocatalytic kinetics

The photocatalytic degradation kinetics of MB over Ca_2_Fe_2_O_5_ at different catalyst dosages were analyzed using the pseudo-first-order kinetic model, which is commonly described by the Langmuir–Hinshelwood equation:7
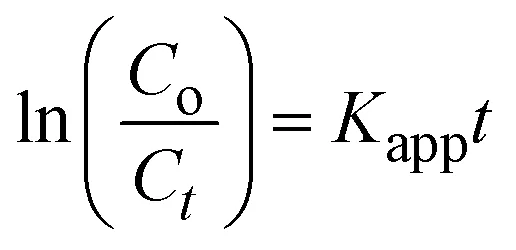
where *C*_o_ and *C*_*t*_ are the initial and instantaneous MB concentrations, respectively, *k* is the apparent first-order rate constant (min^−1^), and *t* is the irradiation time.

As shown in Fig. 13, excellent linear relationships were obtained for all catalyst dosages, with correlation coefficients (*R*^2^) ranging from 0.9993 to 0.9999, indicating that the photocatalytic degradation of MB follows a pseudo-first-order kinetic model. The apparent reaction rate constant increased progressively from 0.00441 min^−1^ for 0.05 g L^−1^ to 0.00860 min^−1^ for 1 g L^−1^, confirming that increasing the catalyst dosage accelerates the photocatalytic degradation process. This enhancement can be attributed to the larger number of available active sites and the increased generation of photogenerated electron–hole pairs and reactive oxygen species, which promote the oxidation of MB molecules. However, the increase in the reaction rate constant becomes less pronounced at higher catalyst dosages, suggesting that excessive catalyst loading does not proportionally improve the photocatalytic activity. This behavior is consistent with the light-screening and light-scattering effects, which reduce light penetration into the suspension and limit the effective utilization of incident photons. Overall, the kinetic analysis confirms that 1 g L^−1^ is the optimum catalyst dosage among the investigated conditions, providing the highest apparent reaction rate and the best photocatalytic performance.

**Fig. 13:**
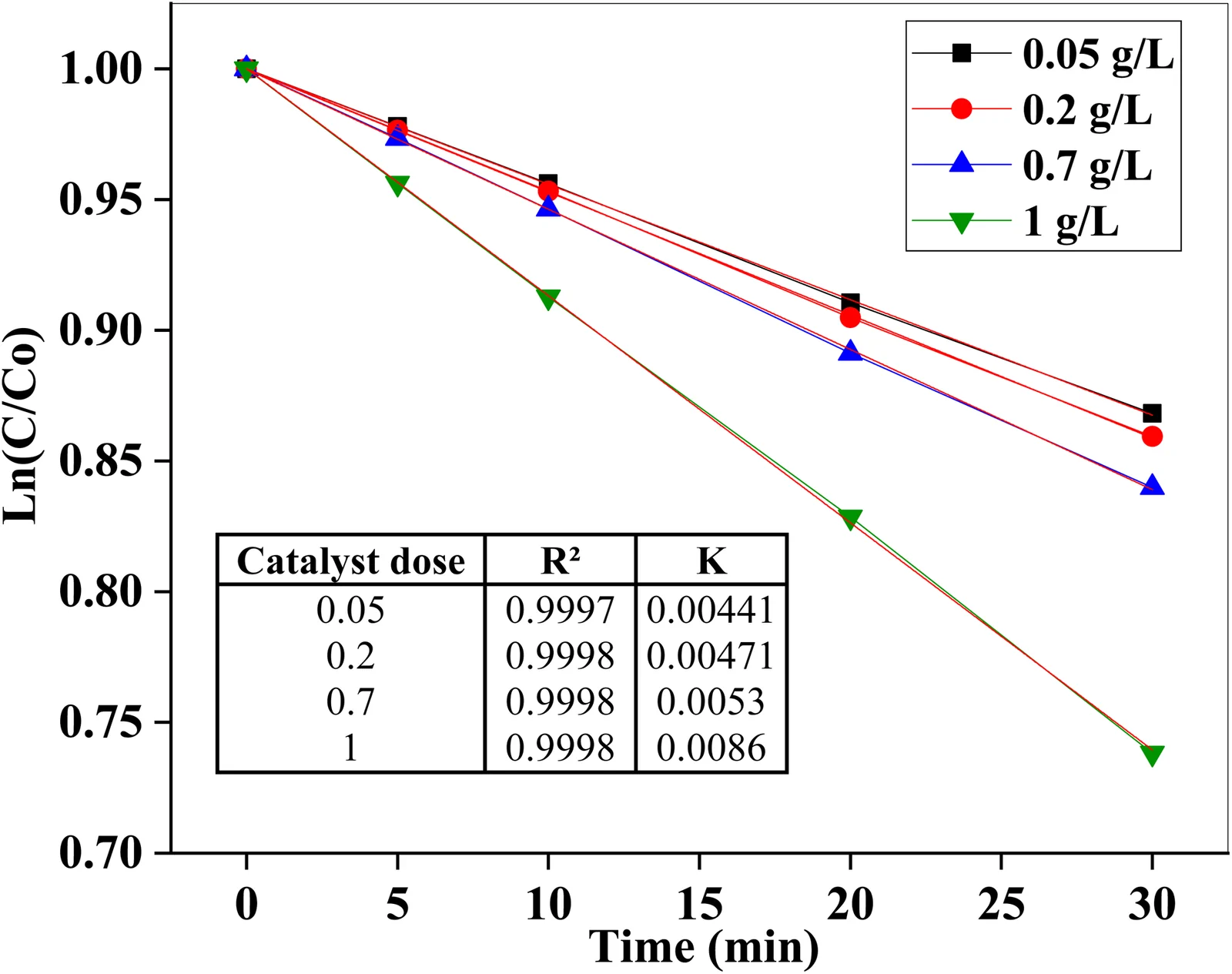
Pseudo-first-order kinetic plots (ln(*C*/*C*_o_) *versus* irradiation time) for MB degradation over Ca_2_Fe_2_O_5_ at different catalyst dosages under natural solar irradiation at natural pH (≈6). The inset summarizes the apparent rate constants (*k*) and correlation coefficients (*R*^2^).

### 4.3 Catalyst reusability

Catalyst reusability is an important criterion for assessing the practical applicability of photocatalysts from both economic and environmental perspectives. Reusing the catalyst reduces material consumption, lowers operating costs, and minimizes waste generation, thereby contributing to the development of more sustainable photocatalytic processes.^56,57^ To evaluate the reusability of Ca_2_Fe_2_O_5_, the recovered photocatalyst was reused in two successive photocatalytic degradation cycles of 10 mg per L methylene blue (MB) under identical experimental conditions. As shown in Fig. 14(a), the photocatalytic activity gradually decreased during the second cycle. The degradation efficiency decreased from approximately 79% in the first cycle to 52% in the second cycle, corresponding to a loss of about 27% (Fig. 14(b)). This decline may be attributed to the partial blockage of active sites by adsorbed reaction intermediates and/or a slight loss of catalyst during the recovery, washing, and handling procedures.^58^ Nevertheless, the catalyst retained a significant photocatalytic activity after the second cycle, demonstrating acceptable stability under the investigated conditions. It is noteworthy that the recovered Ca_2_Fe_2_O_5_ was reused without any chemical regeneration treatment. Combined with the use of natural solar irradiation, this simple reuse procedure further enhances the environmental friendliness and potential economic viability of the photocatalytic process.^59^ Overall, these results demonstrate that Ca_2_Fe_2_O_5_ combines good photocatalytic activity with satisfactory stability and reusability under natural solar irradiation, highlighting its potential for sustainable wastewater treatment applications.

**Fig. 14:**
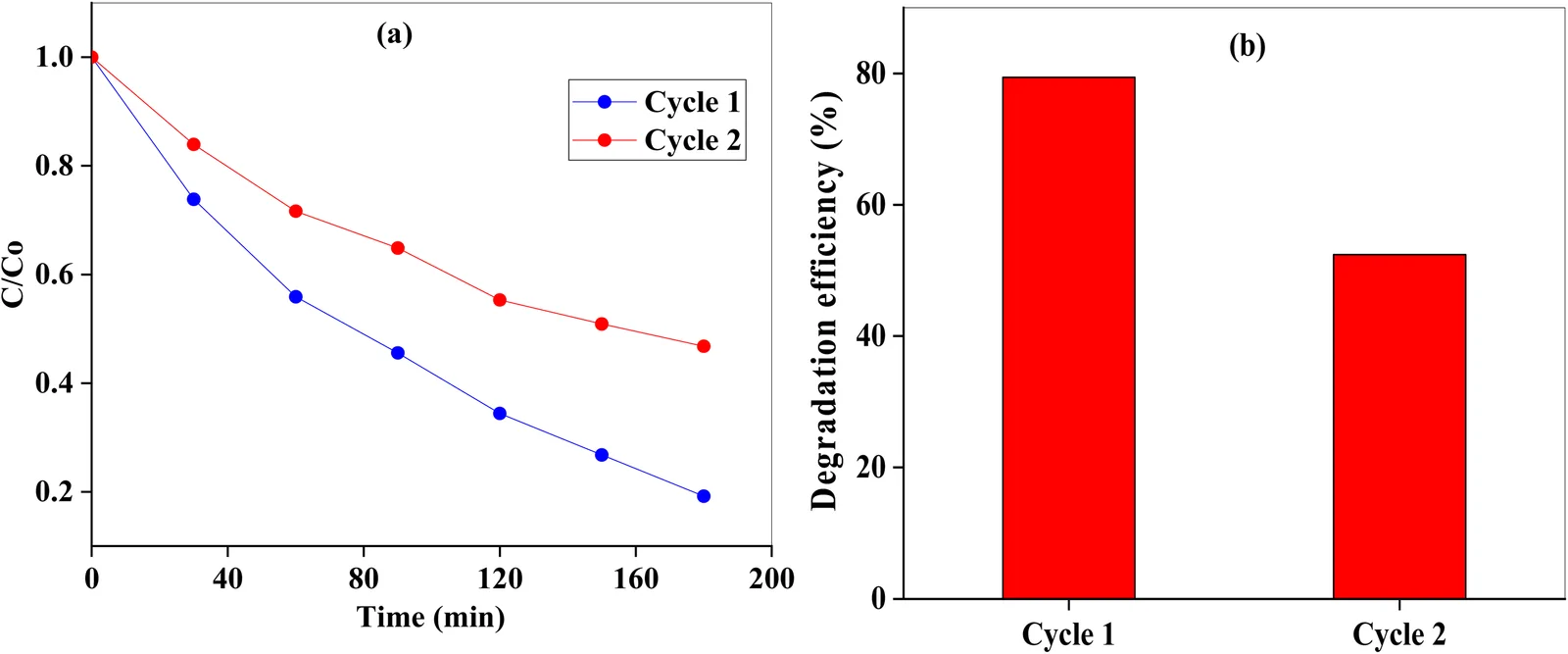
Reusability of the Ca_2_Fe_2_O_5_ photocatalyst for MB degradation: (a) *C*/*C*_o_*versus* irradiation time and (b) degradation efficiency after 180 min (pH ≈ 6, [MB]_0_ = 10 mg L^−1^, catalyst dosage = 1 g L^−1^).

## 5 Conclusion

In this study, brownmillerite Ca_2_Fe_2_O_5_ was successfully synthesized *via* the nitrate route and comprehensively characterized using structural, morphological, surface, optical, electrical, electrochemical, and photocatalytic techniques. X-ray diffraction, SEM-EDX, and XPS analyses confirmed the successful formation of a chemically pure and well-crystallized brownmillerite phase with homogeneous elemental distribution and the expected surface chemical states (Ca^2+^, Fe^3+^, and lattice oxygen). The layered crystal structure, together with the hierarchical porous morphology, is expected to facilitate charge transport and provide abundant accessible surface-active sites. Optical investigations revealed a narrow band gap of approximately 1.92 eV, enabling efficient visible-light absorption, while electrical measurements indicated a low activation energy (0.31 eV), suggesting favorable charge transport. Electrochemical analyses, including Mott–Schottky measurements, cyclic voltammetry, and electrochemical impedance spectroscopy, confirmed the p-type semiconducting nature of Ca_2_Fe_2_O_5_, with a flat-band potential of +0.21 V *vs.* SCE, good electrochemical stability of the Fe^3+^/Fe^2+^ redox couple, and enhanced charge transfer under illumination.

The photocatalytic performance of Ca_2_Fe_2_O_5_ was successfully demonstrated through the degradation of methylene blue under natural solar irradiation. The photocatalytic activity was strongly influenced by the initial solution pH and catalyst dosage, with the highest degradation efficiency (79%) achieved at natural pH (≈6) using a catalyst dosage of 1 g L^−1^. The degradation process followed an apparent pseudo-first-order kinetic model, confirming the efficient utilization of photogenerated charge carriers for the generation of reactive oxygen species (˙OH and O_2_˙^−^) responsible for methylene blue degradation. Furthermore, the catalyst retained satisfactory photocatalytic activity after two successive reuse cycles, demonstrating good stability and reusability under the investigated conditions. Overall, the combination of visible-light absorption, favorable electrical conductivity, hierarchical porous morphology, efficient charge separation, good electrochemical properties, and promising photocatalytic performance highlights brownmillerite Ca_2_Fe_2_O_5_ as a promising visible-light-responsive photocatalyst for sustainable solar-driven wastewater treatment applications.

## Conflicts of interest

There are no conflicts to declare.

## Data Availability

The data supporting the findings of this study are available within the article and provide all the information necessary to support the conclusions presented.

## References

[cit1] Saib F., Laoui F. M., Aoudjit L., Touahra F., Rekhila G., Lerari D., Azoudj Y., Chebout R., Bachari K., Trari M., Abdi A. (2024). Opt. Mater..

[cit2] Saib F., Mekiri M., Bellal B., Chibane M., Trari M. (2017). Russ. J. Phys. Chem. A.

[cit3] Saib F., Bellal B., Trari M. (2017). Mater. Sci. Semicond. Process..

[cit4] Saib F., Touahra F., Azoudj Y., Chebout R., Lerari D., Bachari K., Abdi A., Trari M. (2022). J. Solid State Electrochem..

[cit5] Saib F., Özel F., Sarılmaz A., Mahroua O., Bellal B., Trari M. (2019). Mater. Sci. Semicond. Process..

[cit6] Okpara E. C., Wojuola O. B., Fayemi O. E., Oyewo O. A., Onwudiwe D. C. (2022). J. Inorg. Organomet. Polym. Mater..

[cit7] Nakhowong R. (2015). Mater. Lett..

[cit8] Radhika N. P., Malini S., Raj K., Anantharaju K. S., Shylaja K. R., Appaji A. (2023). Heliyon.

[cit9] Lee S. J. , Kim D. S. and Kim D. K., in 35th International Conference and Exposition on Advanced Ceramics and Composites, The American Ceramic Society, The American Cermic Society's Engineering, 2011

[cit10] Sundriyal S. K., Sharma Y. (2019). ECS Meet. Abstr..

[cit11] Benallal S., Boumaza S., Brahimi R., Trari M. (2022). J. Solid State Electrochem..

[cit12] Benallal S., Boumaza S., Brahimi R., Trari M. (2022). Int. J. Hydrogen Energy.

[cit13] Christy M., Choi S., Kwon J., Jeong J., Paik U., Song T. (2025). Small Sci..

[cit14] Lucinski T., Markiewicz E., Andrzejewski B., Pugaczowa-Michalska M., Chybczynska K., Tran V. H., Idczak R., Skokowski M. P., Chrunik M., Koscielska B. (2025). Acta Phys. Pol. A.

[cit15] Liu L., Zhang C., Chen Z., Luo Z., Zhou H., Wang J., Cao X., Chen Z. (2024). Ceram. Int..

[cit16] Arif M., Gassoumi A., Alharbi F. F., Saddeek S., Abo-Dief H. M., Kumar A. (2026). J. Inorg. Organomet. Polym. Mater..

[cit17] Bhowmik S., Karki S. B., Ramezanipour F. (2025). Chem. Phys..

[cit18] Long T., Song D., Zhou Y., Yu X., Wang X., Li C., Chen H., Li G., He F. (2025). ACS Appl. Mater. Interfaces.

[cit19] Vavilapalli D. S., Srikanti K., Mannam R., Tiwari B., Mohan Kant M., Rao M. S. R., Singh S. (2018). ACS Omega.

[cit20] Fisher C. A. J., Islam M. S. (1999). Solid State Ionics.

[cit21] Zhong Y., Shih K., Diao Z., Song G., Su M., Hou L., Chen D., Kong L. (2021). Chem. Eng. J..

[cit22] Singh G., Ubhi M. K., Jeet K., Singla C., Kaur M. (2023). Processes.

[cit23] Irshad M., tul Ain Q., Zaman M., Aslam M. Z., Kousar N., Asim M., Rafique M., Siraj K., Tabish A. N., Usman M., ul Hassan Farooq M., Assiri M. A., Imran M. (2022). RSC Adv..

[cit24] Li Z., Yan T., Fang X. (2023). Nat. Rev. Mater..

[cit25] Schichtl Z. G., Carvalho O. Q., Tan J., Saund S. S., Ghoshal D., Wilder L. M., Gish M. K., Nielander A. C., Stevens M. B., Greenaway A. L. (2025). Chem. Rev..

[cit26] Xi Z., Liu M. (2025). npj Mater. Sustainability.

[cit27] De Cian L., Díez-García M. I., Goldman B., Sivula K. (2025). RSC Appl. Interfaces.

[cit28] Wang Y., Xu F., Sun L., Li Y., Liao L., Guan Y., Lao J., Yang Y., Zhou T., Wang Y., Li B., Zhang K., Zou Y. (2022). RSC Adv..

[cit29] Devi S., Aarushi N., Tyagi S. (2022). RSC Adv..

[cit30] Balakrishnan A., Flora R. M. N., Ilyas M., Rajamohan N. (2026). J. Mater. Chem. A.

[cit31] Vavilapalli D. S., Peri R. G., Sharma R. K., Goutam U. K., Muthuraaman B., Ramachandra Rao M. S., Singh S. (2021). Sci. Rep..

[cit32] Chakir A., Aqdim S., Mehdaoui B., El Bouari A. (2024). Eur. Phys. J. B..

[cit33] Curtis E. M., Auckett J. E., Evans I. R. (2025). CrystEngComm.

[cit34] Armendáriz S. F., Amézaga-Madrid P., Olive-Méndez S. F., Espinosa-Magaña F., Lardizábal-Gutiérrez D. (2025). Opt. Mater..

[cit35] Bashiri F., Khezri S. M., Kalantary R. R., Kakavandi B. (2020). J. Mol. Liq..

[cit36] Haynes W. M. , CRC Handbook of Chemistry and Physics, CRC Press, Boca Raton, FL, 2010

[cit37] Brockner W., Ehrhardt C., Gjikaj M. (2007). Thermochim. Acta.

[cit38] Wieczorek-Ciurowa K., Kozak A. J. (1999). J. Therm. Anal. Calorim..

[cit39] Saib F., Touahra F., Azoudj Y., Chebout R., Lerari D., Bachari K., Abdi A., Trari M. (2022). J. Solid State Electrochem..

[cit40] Asaei S., Norouzbeigi R. (2021). Ceram. Int..

[cit41] Ismael M. (2021). Sol. Energy Mater. Sol. Cells.

[cit42] Newbury D. E., Ritchie N. W. M. (2013). Scanning.

[cit43] Dhankhar S., Gupta K., Bhalerao G., Shukla N., Chandran M., Francis B., Tiwari B., Baskar K., Singh S. (2015). RSC Adv..

[cit44] Moulder J. F. , Stickle W. F., Sobol P. E. and Bomben K. D., There Is No Corresponding Record for This Reference, 2000, 232–233

[cit45] Seah M. P. , Briggs D. and Grant J. T., Surface Analysis by Auger and X-Ray Photoelectron Spectroscopy, 2003, 167–189

[cit46] Saib F., Abdi A., Haouchine L. A., Touahra F., Laoui F. M., Zemmache S., Lerari D., Bachari K., Trari M. (2025). Polym. Bull..

[cit47] Shaula A. L., Pivak Y. V., Waerenborgh J. C., Gaczyñski P., Yaremchenko A. A., Kharton V. V. (2006). Solid State Ionics.

[cit48] Shukla A., Gaur N. K., Ghosh P. (2020). Appl. Surf. Sci..

[cit49] Asenath-Smith E., Lokuhewa I. N., Misture S. T., Edwards D. D. (2010). J. Solid State Chem..

[cit50] Vuong J., Derakhshan S., Tavassol H. (2024). ACS Mater. Au.

[cit51] Phan T. L., Tho P. T., Tran N., Kim D. H., Lee B. W., Yang D. S., Thiet D. V., Cho S. L. (2018). J. Electron. Mater..

[cit52] Vavilapalli D. S., Qin L., Palisaitis J., Rosen J. (2024). npj Clean Water.

[cit53] Hassan F., Talami B., Almansba A., Bonnet P., Caperaa C., Dalhatou S., Kane A., Zeghioud H. (2024). ChemEngineering.

[cit54] Tawfik A., Alalm M. G., Awad H. M., Islam M., Qyyum M. A., Al-Muhtaseb A. H., Osman A. I., Lee M. (2022). Environ. Chem. Lett..

[cit55] Saha H., Dastider A., Ferdous Anik M. J., Mim S. R., Talapatra S., Das U., Jamal M., Billah M. M. (2024). Results Mater..

[cit56] Karim A. V., Krishnan S., Shriwastav A. (2022). J. Indian Chem. Soc..

[cit57] Almansba A., Kane A., Nasrallah N., Wilson J. M., Maachi R., Lamaa L., Peruchon L., Brochier C., Amrane A., Assadi A. A. (2021). J. Photochem. Photobiol., A.

[cit58] Hsu C. Y., Mahmoud Z. H., Abdullaev S., Ali F. K., Ali Naeem Y., Mzahim Mizher R., Morad Karim M., Abdulwahid A. S., Ahmadi Z., Habibzadeh S., kianfar E. (2024). Case Stud. Chem. Environ. Eng..

[cit59] Djedouani D., Almansba A., Boudjemia M., Ouslimani N., Rechedi Y. (2026). J. Indian Chem. Soc..

